# The Quantitative Basis of the *Arabidopsis* Innate Immune System to Endemic Pathogens Depends on Pathogen Genetics

**DOI:** 10.1371/journal.pgen.1005789

**Published:** 2016-02-11

**Authors:** Jason A. Corwin, Daniel Copeland, Julie Feusier, Anushriya Subedy, Robert Eshbaugh, Christine Palmer, Julin Maloof, Daniel J. Kliebenstein

**Affiliations:** 1 Department of Plant Sciences, College of Agricultural and Environmental Sciences, University of California - Davis, Davis, California, United States of America; 2 Department of Plant Biology, College of Biological Sciences, University of California - Davis, Davis, California, United States of America; 3 DynaMo Center of Excellence, University of Copenhagen, Frederiksberg, Denmark; Max Planck Institute for Developmental Biology, GERMANY

## Abstract

The most established model of the eukaryotic innate immune system is derived from examples of large effect monogenic quantitative resistance to pathogens. However, many host-pathogen interactions involve many genes of small to medium effect and exhibit quantitative resistance. We used the *Arabidopsis*-*Botrytis* pathosystem to explore the quantitative genetic architecture underlying host innate immune system in a population of *Arabidopsis thaliana*. By infecting a diverse panel of Arabidopsis accessions with four phenotypically and genotypically distinct isolates of the fungal necrotroph *B*. *cinerea*, we identified a total of 2,982 genes associated with quantitative resistance using lesion area and 3,354 genes associated with camalexin production as measures of the interaction. Most genes were associated with resistance to a specific *Botrytis* isolate, which demonstrates the influence of pathogen genetic variation in analyzing host quantitative resistance. While known resistance genes, such as receptor*-*like kinases (RLKs) and nucleotide-binding site leucine-rich repeat proteins (NLRs), were found to be enriched among associated genes, they only account for a small fraction of the total genes associated with quantitative resistance. Using publically available co-expression data, we condensed the quantitative resistance associated genes into co-expressed gene networks. GO analysis of these networks implicated several biological processes commonly connected to disease resistance, including defense hormone signaling and ROS production, as well as novel processes, such as leaf development. Validation of single gene T-DNA knockouts in a Col-0 background demonstrate a high success rate (60%) when accounting for differences in environmental and *Botrytis* genetic variation. This study shows that the genetic architecture underlying host innate immune system is extremely complex and is likely able to sense and respond to differential virulence among pathogen genotypes.

## Author Summary

The innate immune system is an important driver of quantitative resistance among all eukaryotes and is the primary immune system for many multicellular organisms, including plants, fungi, and insects. While some components of the innate immune system are known, many components are still undiscovered. Here we use genome wide association (GWA) mapping in a natural population of the *Arabidopsis thaliana* host against four phenotypically and genetically distinct isolates of the fungal pathogen *Botrytis cinerea* to describe and validate components of the plant innate immune system. Using both the induced production of a known defense compound, camalexin, and lesion area as quantified outputs of the plant innate immune system, we found approximately 2,982 and 3,354 genes associated with quantitative resistance respectively. Genes associated with variation in the plant immune system were largely dependent on the pathogen genotype, indicating the presence of specificity in pathogen detection and response by the host. Associated genes, which were filtered into co-expression networks followed by GO enrichment analysis, showed both known and novel biological processes associated with pathogen response. Validation of associated genes using single gene knockouts in a common genetic background indicates an ability to validate causal genes with a 60% validation rate.

## Introduction

Our understanding of host/pathogen interactions is largely driven by examples of large-effect, qualitative resistance involving a small subset of host genes focused on detection and signal responses to the pathogen. However, many host/pathogen interactions are predominantly quantitative in nature involving an unspecified number of genes with moderate to small effects involved in detection and response to the attacking pathogen. Quantitative resistance is characteristic of host resistance to endemic pathogens that are ubiquitous within a host’s environment and create a persistent selective pressure on the innate immunity of the host [1]. This constant selective pressure from endemic pathogens prevents the development of highly susceptible host alleles and may canalize the genetic variability of many genes involved in resistance. This differs distinctly from epidemic pathogens that are extremely scarce between outbreaks allowing for the development of resistance genes with large-effect alleles that can spread throughout the host population [2,3]. The continuous nature of quantitative resistance suggests that there may be alleles at a number of genes that can provide long-term, durable resistance to these pathogens. However, this lack of large effect natural alleles and the large number of genes that are thought to be involved in quantitative resistance complicates our understanding of involved biological functions and pathways.

The resistance mechanisms utilized by the host may also depend upon the lifestyle of the pathogen. Quantitative resistance is often seen in response to necrotrophic pathogens, such as *Aspergillus fumigatus* in humans [4,5], southern leaf blight (*Bipolaris maydis*) in maize [6], and pitch canker (*Fusarium circinatum*) in loblolly pine [7]. However, there have been very few quantitative resistance loci actually cloned and the underlying genetic architecture or molecular mechanisms associated with this natural variation is relatively hidden [6]. Comparatively, qualitative resistance is commonly observed in specialist, biotrophic pathogens where host individuals can easily be categorized into susceptible and resistant genotypes for a single strain of the pathogen. Examples include *Vibrio cholerae* in mammals [8], *Xanthomonas oryzae* in rice [9,10], and *Pseudomonas syringae* in *Arabidopsis* and tomato [11,12]. The large-effect polymorphisms involved with qualitative resistance lend themselves nicely to investigation through Mendelian genetics allowing for the identification of the individual causative genes. Currently, the vast majority of qualitative resistance genes identified using natural allelic variants in *Arabidopsis* have been either direct or indirect receptor proteins, including receptor-like kinases (RLKs) and nucleotide-binding site leucine-rich repeat proteins (NLRs) [13–18]. The QTL studies leading to the cloning of these genes typically found large and small effect loci but for experimental reasons, focused solely on the cloning of large effect loci, leaving the molecular identity of the small effect loci uncovered. In contrast, naturally variable alleles in downstream components of qualitative resistance, such as mitogen-activated protein kinases, transcription factors, and proteases/lipases, have largely not been found underlying the large effect qualitative loci. These downstream components have predominantly been identified through the use of careful molecular assays, including mutant screens, protein co-immunoprecipitations, and yeast one/two hybrid assays. Expression QTL analysis investigating the impact of major effect R genes showed that these large effect loci function via a vast array of downstream loci, but it was not clear if genetic variation in these downstream loci also contributed to disease resistance [19–22]. Thus, while it is clear that variation in qualitative resistance works through the upstream receptors and signaling pathways, it is unclear whether these loci and/or pathways play a key role in the variation of quantitative resistance. To empirically measure quantitative resistance and identify the genes that control variation in this resistance, we are using the endemic plant fungal pathogen *Botrytis cinerea* to conduct a genome-wide association (GWA) study with the model host plant *Arabidopsis thaliana*. *Botrytis cinerea* is a necrotrophic fungal pathogen of plants that is found on every continent with a temperate climate and is thought to have a pan-global natural range prior to human agriculture [23]. This endemic pathogen has a large amount of standing genetic variation in all currently studied populations [24–28] and has an extremely large host range that spans non-vascular and seedless plants to most true eudicots [29–31]. There are currently no known large-effect qualitative resistance alleles identified in any plant for resistance to *B*. *cinerea* and quantitative studies in structured mapping populations have shown that resistance is highly polygenic with significant epistasis that is highly dependent on genetic variation between *Botrytis* isolates [32–34]. Thus, *B*. *cinerea* is a highly useful model pathogen that allows us to both identify the underlying mechanisms of quantitative resistance in innate immunity and to assess how pathogen genetics influences these loci.

For the plant host, we are using the model plant *Arabidopsis thaliana* to measure quantitative resistance to this endemic fungal necrotrophic pathogen. *Arabidopsis thaliana* has already provided a wealth of information for understanding host resistance within pathology including some loci identified to control resistance to a variety of pathogens via natural alleles as well as forward and reverse mutant screens [35–37]. Specifically, the use of natural variation in the host has allowed the identification of a small set of novel genes involved with *B*. *cinerea* resistance including an RLK specific for mannitol perception [36,38,39]. To gain a broader view of quantitative resistance and the potential mechanisms involved, we are utilizing the rich genetic resources of natural variation across *Arabidopsis* accessions [40] to conduct a GWA study and identify individual genes and biological processes associated with resistance to *B*. *cinerea*. Natural populations used in GWA studies have several advantages over structured breeding populations, such as increased allelic variants per locus and higher recombination between loci allowing for finer mapping [41]. However, these populations can be limited if natural selection imparts a strong structure on the underlying allelic variation such that some variants or combinations are missing from the population. GWA has already been proven to be effective by recapitulating results from previous qualitative resistance studies [42] and by identifying new genes controlling defense mechanisms within *Arabidopsis* [43]. GWA has also been applied to a few non-model or genomically complex host species [6,7] to identify novel components of quantitative resistance, many with unknown function. However, these studies were unable to validate if these genes affect quantitative resistance. By working with the model plant host *A*. *thaliana*, we can combine GWA hits with the vast store of gene expression studies to help identify co-expression modules that are involved with quantitative resistance and provide putative function to genes with unknown function as we have previously done for defense metabolism [43].

In this study, we measured quantitative resistance in a collection of natural *A*. *thaliana* accessions individually infected with one of four phenotypically and genetically diverse *B*. *cinerea* isolates. We quantified resistance by both lesion size and camalexin production as a quantitative biochemical marker of the plant’s defense response. Our study shows that quantitative resistance can involve thousands of genes linking to a wide variety of cellular processes ranging from hormone signaling and reactive oxygen signaling that have previously been associated with disease resistance to development and RNA processing, which to our knowledge, has not been associated with disease resistance. The data also suggest that there is a hierarchical nature to related disease phenotypes with some genes contributing to both lesion development and defense response while others appear to be specific to one aspect of quantitative resistance. In addition, our study also illustrates that quantitative resistance of the plant host is highly specific to the pathogen’s genotype. Thus, mapping for quantitative resistance can provide very different answers depending on which isolate is used and suggests that even quantitative resistance will have difficulty in providing durable resistance within host populations.

## Results

### Selection of the *Arabidopsis* Population

*Arabidopsis* accessions have a wide range of flowering times that can affect the ontogenic status of the plant and influence plant/pathogen interactions [42,44]. Thus, we wanted to choose our GWA population to minimize any ontogenic influence on quantitative resistance while still maintaining genetic diversity. As such, we filtered a population of 241 accessions to a collection of 96 individuals based on similar flowering time (63.1 ± 0.95 (s.e.) days to flowering under short-day conditions [42]). This filtering should both alleviate issues with differential rate of development across the accessions and minimize any potential influence of flowering time in our mapping. Importantly, this collection includes several natural accessions that are the parents of defined breeding populations that have been screened for resistance to *B*. *cinerea* (i.e. Bay-0, Sha-0, and Le*R-*1) [33,34].

**Fig 1:**
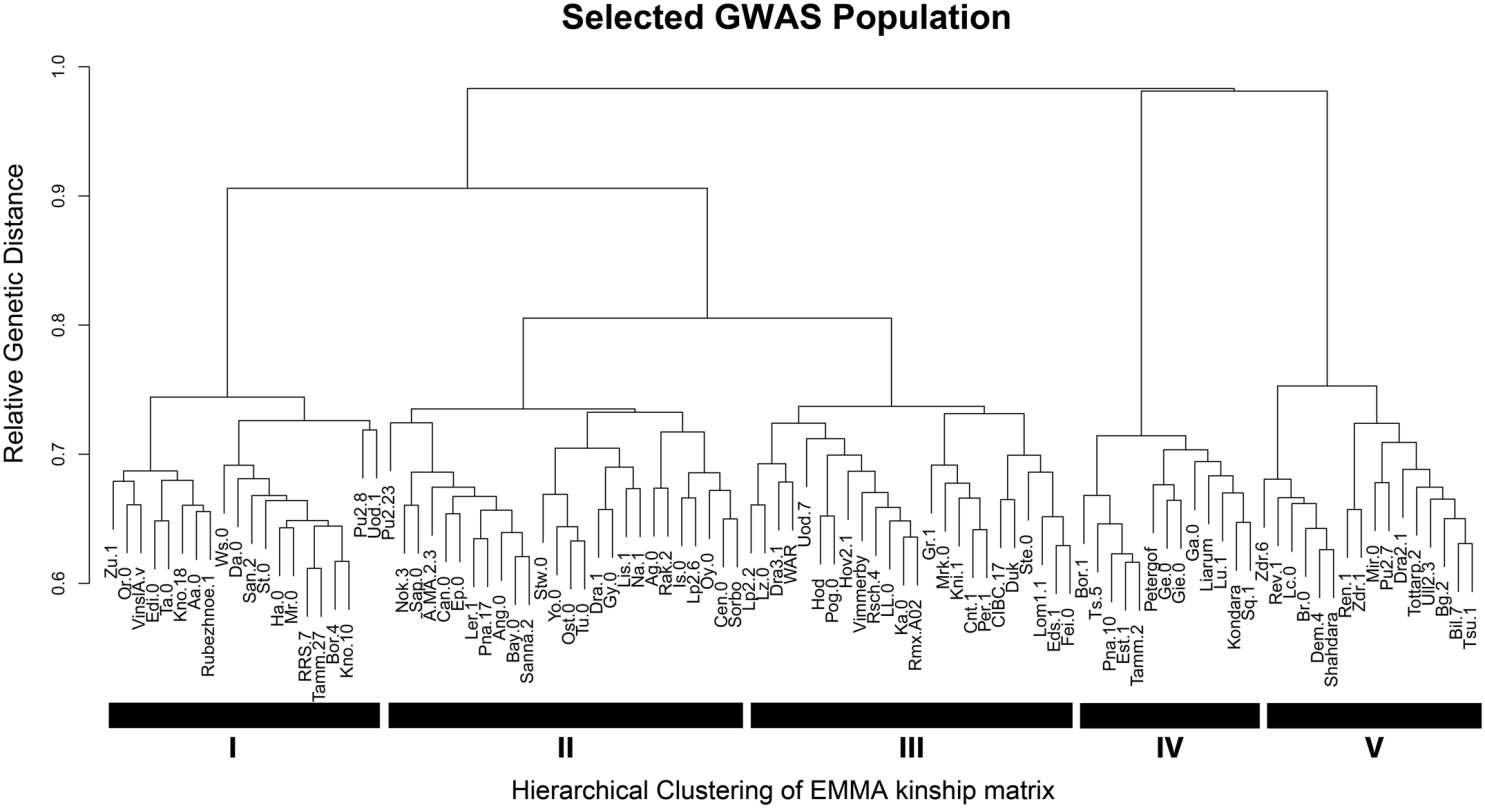
Kinship matrix dendrogram for the *Arabidopsis* mapping population. Dendrogram of the EMMA kinship matrix for selected *Arabidopsis thaliana* accessions used for GWA mapping. Accessions were filtered for common flowering time (63.1 ± 0.95 (s.e.) days to flowering) to reduce effects from ontogeny. These accessions fall into approximately five major groups (I-V) as illustrated.

Another potential concern for GWA mapping within a natural population is the effect of population structure and rare alleles that result in spurious associations between the phenotype and an allele [45–47]. To assess the level of population structure in our population, we performed a hierarchical clustering analysis of the kinship matrix for 215k SNPs for both the 241 accessions and the ontogenically-filtered collection of 96 accessions. The analysis showed that by simply filtering on flowering time in a growth chamber, we were able to remove the majority of large genetic outliers as well as the majority of population structure (Fig 1 and S1 Fig). Further, while the remaining accessions cluster into five major genomic subgroups, these subgroups do not appear to cluster to a specific geographical region (S2 Fig). While growth chamber measured flowering time is correlated with geography, there is still significant variation within each region and our filtering appears to have removed the extreme flowering individuals and chosen a subpopulation that encapsulates the majority of the geographic range of the species. The final population contains a total of 115,301 SNPs with a minor allele frequency > 0.2 spanning a total of 19,352 genes tested (S1 Table). This population selection should reduce the influence of correlated ontological phenotypes and potentially the false positive rate from rare alleles and population structure, which we tested empirically and describe below (Fig 1).

### Phenotypic Structure of Lesion Area and Camalexin

To investigate the basis of quantitative resistance and how genetic variation in the host and pathogen influence the ability to identify the underlying loci, we infected the above 96 accessions with four phenotypically diverse *Botrytis cinerea* isolates chosen based on their diversity in previous studies [33,48–51] using a randomized complete block design with four replicates in each of two independent experiments. We measured lesion size at 72 hours post infection using a digital image analysis pipeline developed in the R statistical environment using the EBImage and CRImage packages [52,53]. Additionally, all leaves were collected at 72 hours to be assayed for camalexin, a key phytoalexin providing defense against *Botrytis*, via HPLC to provide a measure of plants quantitative defense response to the interaction. F-tests derived from separate generalized linear models (GLMs) for both camalexin content and lesion size revealed that there was a statistically significant interaction between the genotype of the plant host and genotype of the isolate (Table 1). This indicates that quantitative disease resistance within the host is highly isolate dependent. In contrast, the effect of the experimental replicate, individual plant, and interactions of treatment:plant were non-significant for camalexin showing that the biological effects drive most of the phenotypic variance for this experiment (S2 Table). While there was a significant effect of experiment and individual plant on lesion size, we were able to account for this using the model (Table 1). We chose to analyze each quantitative resistance in response to each pathogen genotype separately since both traits show a statistical significant interaction between the host and pathogen genotypes. This should both simplify the analysis and avoid confounding the results across the isolates.

**Table 1:** F-Tables of GLM for camalexin and lesion area. F-tables from the whole experiment GLM for each phenotype using type II sums of squares. Significance was determined as follows: 0 ‘***’ 0.001 ‘**’ 0.01 ‘*’ 0.05 ‘.’ 0.1 ‘ ‘ 1. Both phenotypes show highly significant interaction between the host and pathogen genotype. All future results were split per isolate as a result.

	Sum of Squares	Df	F-value	P-value
**Camalexin**				
Experiment	161	1	0.1199	0.72919
Treatment	743877	1	552.557	< 2e-16***
Accession	166169	96	1.2857	0.03542*
Plant	917929	623	1.0945	0.08132
Treatment:Isolate	3691855	3	914.111	< 2e-16***
Treatment:Accession	33876	96	0.2621	1
Treatment:Plant	271312	623	0.3235	1
Treatment:Accession:Isolate	878209	288	2.2651	< 2e-16***
Residuals	2462283	1829		
**Lesion Area**				
Experiment	1224	1	9.3026	0.00232**
Isolate	333466	3	844.511	< 2.2e-16***
Accession	25273	96	2.0001	7.39E-08***
Plant	179962	623	2.1947	< 2.2e-16***
Isolate:Accession	57552	288	1.5183	4.20E-07***
Residuals	246131	1870		

**Fig 2:**
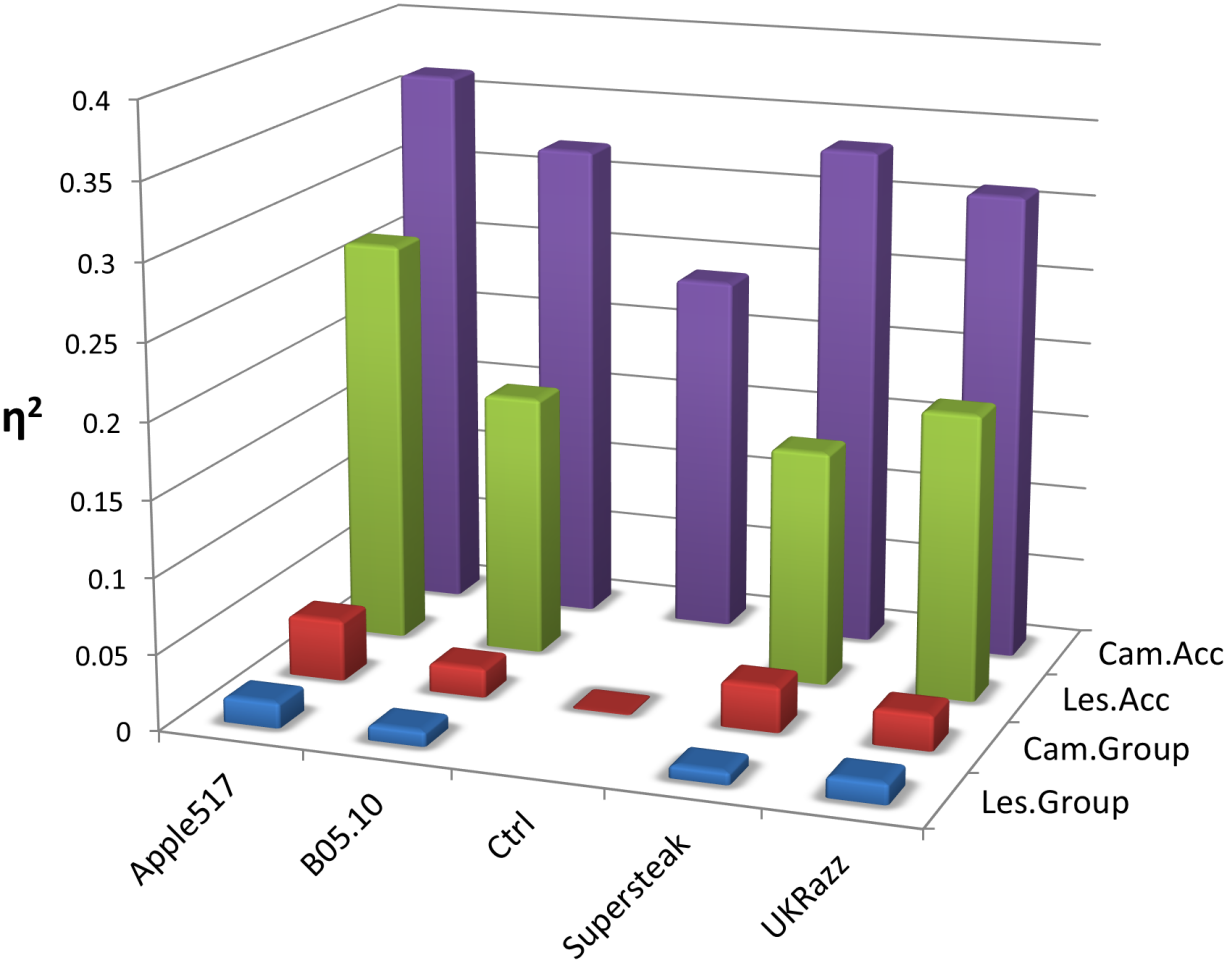
Proportion of variance (η^2^) for genotypic group vs accession genotype. Bar graphs illustrating the proportion of variance (η^2^) explained by the five major groups (I-V) versus the individual accessions. Variance was determined using a standard guassian linear model where Phenotype = Experiment + Experiment(Plate) + Group + Group(Accession) for each *Botrytis* isolate.

Another concern for genetic mapping in natural populations is the ability of population structure to drive false positive associations. To address this concern we conducted additional GLMs for both camalexin and lesion area for each isolate separately to determine how much phenotypic variance is attributable to the residual population structure. The F-tests revealed that the residual population structure accounted for a significant amount of variation for camalexin in response to all four isolates but was not significant for lesion area (S2 Table). Importantly, however, the proportion of variance (η^2^) attributable to the phylogenetic group was on average only 7% of the level of phenotypic variation attributable to differences among the accessions (S2 Table, Fig 2). Model corrected means for genetic mapping were derived from this model using this population to remove potential effects derived from residual population structure. Notably, the distribution of these model corrected means were largely unimodal with only slight skewing that did not require transformation given the large sample size (S3 and S4 Figs). This approach should help reduce false positive associations attributable to the general population structure.

To test if there is a relationship between lesion size and camalexin induction across the host/pathogen genotype combinations within this dataset, we calculated Spearman’s rank correlations among the phenotypes. This analysis showed that correlation between camalexin production and lesion area is significant but weakly negative (S5 Fig), as previously found [33,34]. Within this analysis, camalexin production between the different isolates showed a higher correlation across accessions than was observed for lesion size. This suggests that the genetic variation for camalexin response may be more similar across the accessions than is the genetic variation for lesion size. Interestingly, the basal camalexin concentration prior to infection had no correlation with either induced camalexin content or lesion size, indicating that basal levels of camalexin are poor predictors of induced camalexin or disease resistance within this collection of accessions.

### Genome-Wide Association Mapping of Camalexin and Lesion Area

To conduct the GWA mapping and identify individual candidate genes associated with quantitative resistance in *Arabidopsis*, we used the model corrected means from the full experimental model described above in a separate ridge-regression GWA model. Ridge regression has the advantage over single marker procedures, such as EMMA [46], of testing all SNPs in single model by treating each SNP as a random effect and avoids issues with multiple test corrections [54]. This benefit comes at the expense of obtaining a direct p-value for each SNP but does provide estimates of the heteroscedastic effect of each SNP. We calculated a significance effects threshold by randomly permuting the phenotypes among the genotypes 1,000 times and pulling the 99th percentile from the distribution of effects derived from the permutations to identify significant SNPs. Because this permutation maintains relationship of genotypes within the matrix, it maintains any population structure across the permutations and should help to control for spurious effects caused by population structure. The genetic map for the accessions included a total of 115,301 SNPs with a minor allele frequency (MAF) > 0.2 that covered 19,352 unique genes (S1 Table). We identified a total 20,476 SNPs associated with camalexin accumulation and 23,199 SNPs associated with lesion size across the different isolates (S4–S8 Figs and S3 Table). As some SNPs may be false-positive associations due to genetic linkage with the true casual SNP, we defined significantly associated genes as having ≥2 SNPs that crossed with the permuted threshold within the predicted mRNA coding region, similar to the previous approach by our lab [43]. Using this definition, we identified a total of 2,982 genes associated camalexin production and 3,354 genes associated with lesion area (S4–S8 Figs and S3 Table). Approximately one third of the genes identified were found to associate with both camalexin and lesion size. This supports the idea that, while they are related measures of quantitative resistance, there are still differences in the genetic architecture underlying the phenotypes (S9 Fig and S3 Table).

**Fig 3:**
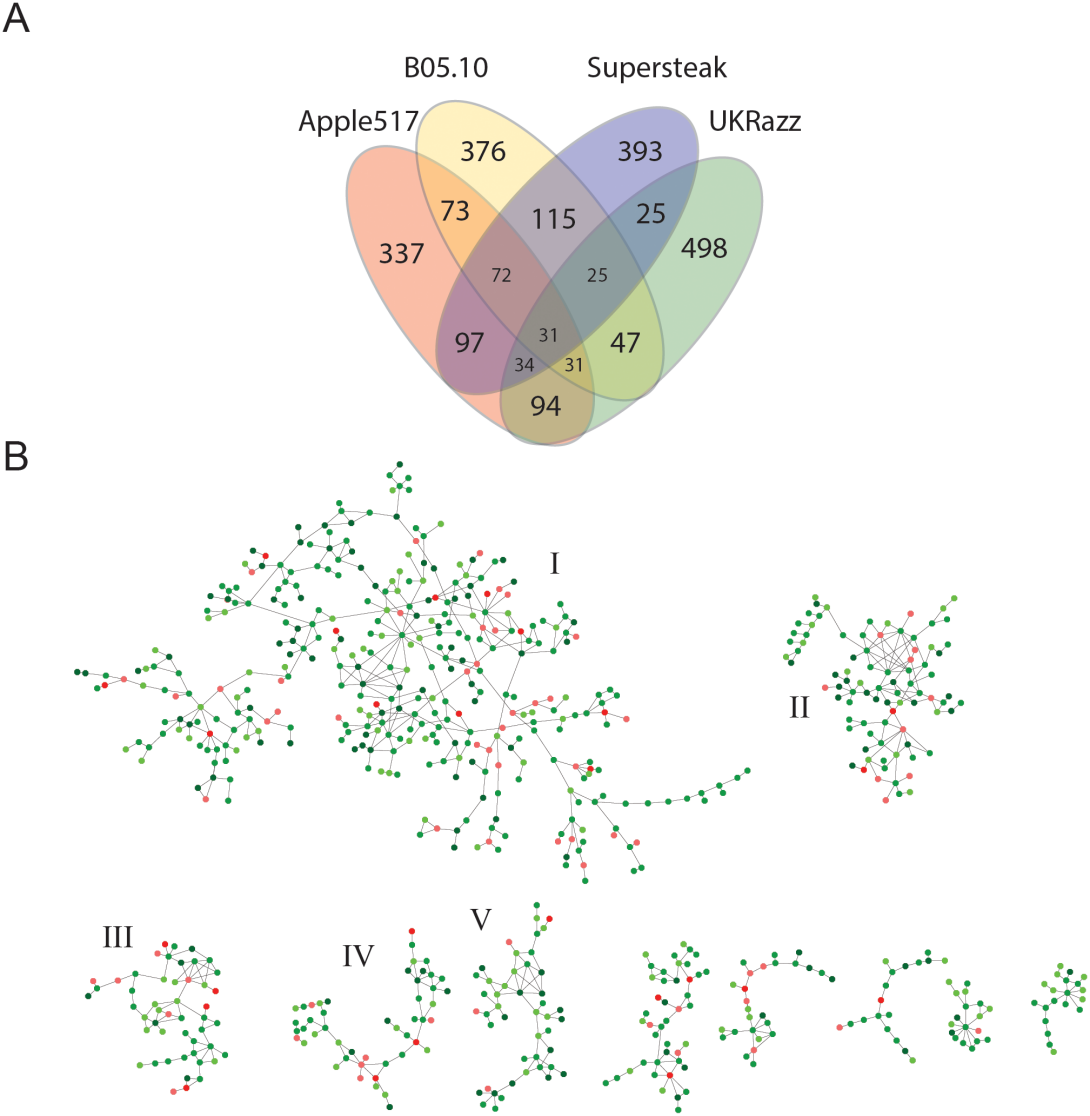
Common genes and co-expression networks found to associate with variation in camalexin production. (A) Venn diagram showing the overlap of candidate genes identified among the four phenotypically diverse isolates for camalexin (ng/mm) accumulation. (B) ATTEDII Co-expression networks for genes *Arabidopsis* significantly associated with camalexin (ng/mm) accumulation across the *Botrytis* isolates. The suggested biological function of the five largest networks was determined via GO enrichment analysis. Putative biological function of the five largest networks are; I) Cell Cycle, Vesicle Trafficking, Protein Turn-over, Jasmonate Signaling, II) Defense Response and Salicylate Signaling, III) Reactive Oxygen Species (ROS) Tolerance, IV) DNA Methylation V) RNA Processing and Cell Wall Modification. Red nodes indicate a gene that was found in at least 3 isolates, dark green nodes indicate a gene was found for 2 isolates and light green indicates 1 isolate.

**Fig 4:**
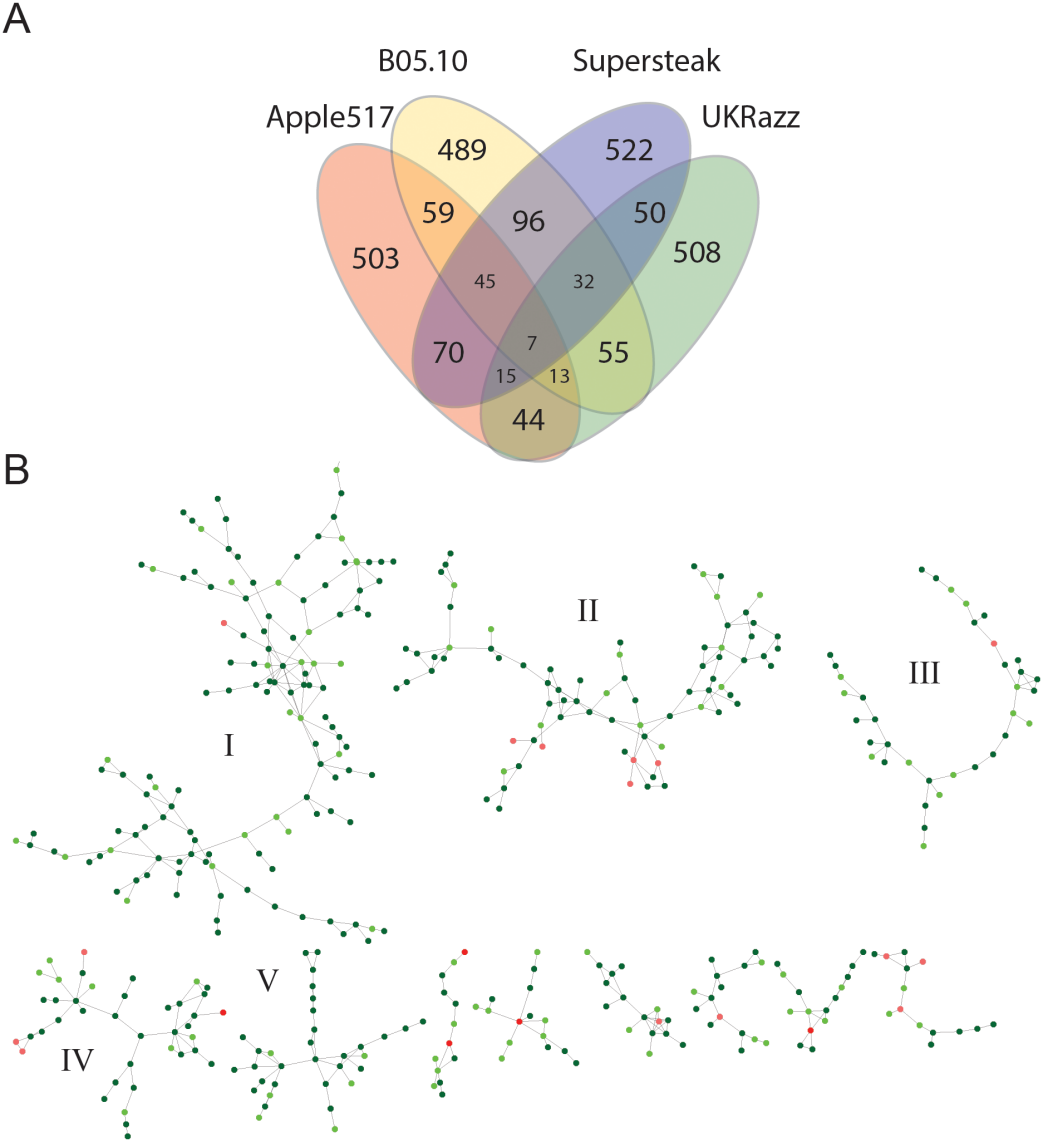
Common genes and co-expression networks found to associate with lesion area. (A) Venn diagram showing the overlap of candidate genes identified among the four phenotypically diverse isolates for lesion area (mm^2^). (B) ATTEDII Co-expression networks for genes *Arabidopsis* significantly associated with lesion size across the *Botrytis* isolates. The suggested biological function of the five largest networks was determined via GO enrichment analysis. Putative biological functions of the five largest networks are; I) Cell Cycle, Gravitropism, and Leaf Development, II) Disease Resistance, III) Amino Acid and Lipid Metabolism, IV) Catabolism and Metabolic Processes, and V) Trehalose and Polysaccharide Metabolism. Red nodes indicate a gene that was found in at least 3 isolates. Red nodes indicate a gene that was found in at least 3 isolates, dark green nodes indicate a gene was found for 2 isolates and light green indicates 1 isolate.

Notably, most of the genes identified as candidates for controlling resistance to the different isolates were unique to the specific isolate with camalexin only having 38 genes in common among the four isolates (Fig 3A and S3 Table) and lesion area having only 9 genes in common (Fig 4A and S3 Table). Of the genes that were common among the isolates, none of these genes are shown to be in common for both camalexin and lesion area, and most are currently understudied with unknown functions (S3 Table). To test if our significance threshold or whether our two-SNP approach is biasing the overlap of candidate gene hits, we utilized different significant thresholds (0.95, 0.975, 0.99 and 0.999) as well as requiring a single SNP or two SNPs and reassessed the presence or absence of overlap amongst the candidate genes. This showed that the pattern of candidate genes showing little overlap across isolates was consistent across all permutation thresholds and SNP calls tested (S11 Fig). The fact that most candidate genes consistently appear unique to a subset or individual isolates at a variety of thresholds supports the idea that quantitative resistance to *B*. *cinerea* is isolate specific and host signaling/response pathways may depend on the different collections of PAMPs and effectors produced by the different pathogen genotypes.

### Most GWA Candidate Genes Are Previously Unlinked to *Botrytis*-Resistance

To test if the GWA approach is identifying novel genes or is largely uncovering genes previously identified as being involved with *B*. *cinerea* resistance, we used a collection of 101 genes previously identified as providing differential resistance to *B*. *cinerea* within *Arabidopsis* when functionally disrupted [55,56]. These studies were largely conducted using a single isolate of *B*. *cinerea* and not collections of diverse genotypes. We then tested how many of these 101 genes known to affect resistance to *B*. *cinerea* within *Arabidopsis* were also found in our collection of GWA candidates. Of these genes, 41 genes did not have two SNPs with a MAF > 0.2 in our population, and therefore could not be identified in our study (S4 Table). Of the remaining 59 known quantitative resistance genes, only 12 genes had two or more significant SNPs associated with either camalexin or lesion area and were usually only identified as a candidate gene in either one or two of the four total isolates (S4 Table). These genes include RLM3, RST1, NPR1, VSP2, ATG2, PAD4, LYK3, COI1, LYK4, Erecta (ER), AXR1, and BOI (S4 Table). Previous work has shown that key resistance regulatory genes that have been found by mutagenesis are likely under purifying selection [57]. The 37 known causal genes that were not identified in the GWAS are likely to have insufficient genetic or functional variation amongst the natural accessions to be found in a GWA approach. Thus, out of the 3,504 genes that we identified as possible candidates for controlling quantitative resistance, only 12 have been previously linked to *B*. *cinerea* resistance.

### R-gene and GO Category Enrichment

A current model of quantitative resistance either directly or indirectly implies that genetic diversity in *R-*genes, including receptor*-*like (RLKs) and Nod-like receptors (NLRs), are the main drivers of quantitative resistance. To address this hypothesis with our list of candidate genes associated with quantitative resistance, we looked for enrichment in associations with genomic lists of potential RLK and NLR *R-*genes [58,59]. Using a hypergeometric test, we found a clear enrichment in NLRs, and to a lesser extent RLKs, for both camalexin and lesion size amongst our candidate genes (S5 Table). A total of 40 *R-*genes were identified in both the camalexin and lesion area mapping. All of these genes are unknown, with the exception of one RLK (NIK3) and three NLR proteins (WRKY19, RLM3, and DAR4) (S5 Table). While these known genes all have been identified in different aspects of disease resistance, RLM3 is the only gene that has been previously characterized as conveying resistance to a broad range of fungal pathogens including *Botrytis* [60]. While there is a significant over*-*representation of these associated *R-*genes, they only account for a small percentage of the total number of associated genes in the study (5.5% of camalexin genes and 6.2% of lesion area genes) (S3 and S5 Tables). Thus, while it appears that some *R-*genes do underpin a component of the measured quantitative resistance phenotype, they do not define the vast majority of genes identified in this study.

To identify what other cellular functions may be involved with quantitative variation in the host innate immune system, we conducted an unbiased gene ontology (GO) enrichment of biological processes using all associated genes (S10 Fig and S6 Table). This analysis revealed that genes associated with variation in camalexin production are enriched in transmembrane transport and cell cycle. Alternatively, genes associated with variation in lesion area are enriched in more diverse biological processes including defense response, oxidation-reduction processes, and nucleic acid mismatch repair mechanisms (S6 Table). Genes associated with lesion area were also enriched in microsporogenesis-related processes, which is likely related to callose deposition as both pollen coats and lesion perimeters show increased callose deposition [61,62]. Both camalexin and lesion area also shared some similar enriched biological processes, which include methylation-dependent chromatin silencing, transmembrane transport, mismatch repair mechanisms and categories related to modification of the cell wall (S10 Fig and S6 Table). Again, these results were unchanged by utilizing different significance thresholds or candidate gene calling methodology. Thus, a GO analysis implicates a variety of cell related processes that are linked to variation in both measures of quantitative resistance, illustrating the complexity of defense response.

### Co-expression Networks

The above results show that natural variation in quantitative resistance involves novel genes far beyond *R-*genes and other known defense genes. Given this multi-locus architecture of quantitative variation in the *Arabidopsis* innate immune system, we worked to identify candidate networks controlling this variation. To do this, we filtered all associated genes through the ATTEDII co-expression database [63,64] to identify potential modules of candidate defense genes that are co-regulated. This filtering could also help reduce the number of false associations from the genetic mapping due to linkage and we have previously utilized this approach to identify genes associated with *Arabidopsis* defense metabolism with a >70% rate of successful validation [43]. Co-expression of two genes was defined as having a mutual rank (MR) < 15 and the putative biological function of co-expression networks was determined using GO-enrichment analysis against all testable genes using our pipeline at multiple statistical thresholds from the GWAS data (0.95, 0.975, 0.99, and 0.999) (S14 Fig). The camalexin and lesion area associated genes produced approximately the same number of networks, 25 and 21 networks with >10 genes respectively, but the camalexin networks were on average larger (36 nodes) than lesion area networks (24 nodes) (Figs 3B and 4B). This is in contrast to the fact that there are more GWA candidate genes for lesion area than for camalexin. Notably, this suggests that the difference in network size between the two phenotypes may be due to the overall transcriptional complexity of the candidate genes. Utilizing the higher permutation thresholds (0.999 or greater) to call candidate genes led to a greatly diminished ability to identify any co-expression networks since too few candidate genes remained at the higher significance thresholds to create meaningful networks. Additionally, lower thresholds appeared to collapse co-expression networks in to large single networks that are difficult to interpret from a biological perspective (S14 Fig). Thus, we chose to continue with the 0.99 permutation threshold analysis.

**Fig 5:**
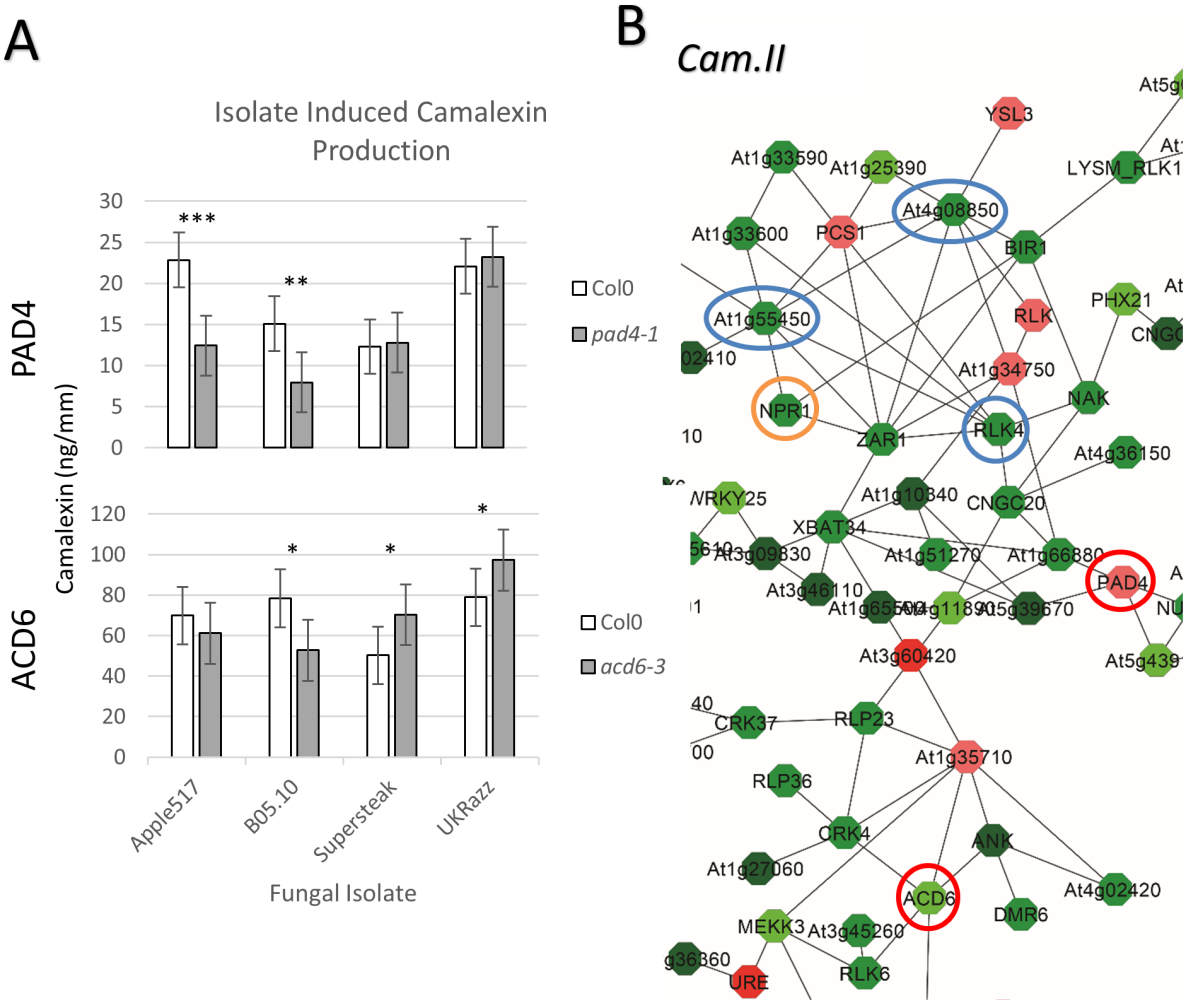
Camalexin validation and network. (A) Bargraphs showing the model corrected means for induced camalexin production of all four isolates on wildtype Col-0 versus *pad4-1* and *acd6-3* mutants. Significance of the difference in camalexin accumulation between Col-0 and the *pad4-1* mutant with each isolate are shown as P< 0.001 ‘***’, 0.001 ‘**’ and 0.01 ‘*’. (B) Magnified images of the Cam.II co-expression network with visible gene names are shown with genes highlighted in the text circled. Single gene knockouts are circled in red while known major defense hormone receptor genes are circled in orange and major hubs are circled in blue.

GO analysis of the five largest co-expression networks for each phenotype identifies a variety of enriched biological functions. The largest network (I) associated with camalexin production was *not* enriched for GO categories in disease resistance but *is* enriched for GO categories connected to cell cycle, vesicle trafficking, and protein/metabolite turnover (Figs 3B and 5A; S7 Table). The two largest hubs within the network are an uncharacterized transducin-like (At3g50590; degree = 12) and an uncharacterized phosphoinositol kinase (At2g1790; degree = 9). The second largest camalexin network (II) is enriched in GO categories typically associated with the plant defense response (Figs 3B and 5B; S7 Table), and includes the salicylate receptor (*NPR1*). Interestingly, NPR1 is proximally located to several large hubs within this network, including the NLR protein *ZAR1* (At3g50950; degree = 8), a SAM-dependent methyl transferase (At1g55450; degree = 8), and an unknown RLK (At4g08850, degree = 8). Camalexin-associated network III is an extremely inter*-*connected network with few well characterized genes, but appears to be involved with production and tolerance of reactive oxygen species (S7 Table). Network IV is a small network (n = 48) that appears to include genes that regulate DNA methylation (S7 Table) and contains two major hubs that are well-studied *Arabidopsis* genes; *ERECTA* (At2g26330, degree = 6) and the histone 2A protein *HTA11* (At3g54560, degree = 6) (S3 Table). Lastly, genes contained in camalexin-associated network V (n = 46) include important enzymes for cell wall formation, including cellulose synthase 10 (At2g25540; degree = 4) and uncharacterized pectin-esterase hub (At5g49180; degree = 7). However, GO enrichment of camalexin network V showed enrichment in RNA processing (S7 Table), which suggests that cell wall modification and RNA processing may be commonly co-regulated. Thus, natural variation in genes involved with a variety of host processes can have quantitative implications on a host’s ability to produce camalexin during pathogen infection.

Co-expression networks of genes associated with lesion area were structurally similar to networks associated with camalexin, but were considerably smaller (S8 Table). The largest network associated with lesion area (I; n = 129 genes) was enriched in GO categories related to gravitropism, cell cycle, and leaf development (Fig 4B; S7 Table), and includes a highly interconnected group of hubs that appear to be involved with RNA/DNA binding and protein degradation (At1g33680, At2g28540, At3g50590, At4g38600, At5g35430, At5g62000). Interestingly, the uncharacterized transducin-like protein (At3g50590) was a major co-expression hub in both the lesion area network I (degree = 9) and the camalexin network I (degree = 12). This indicates that this WD40-repeat protein may play a key role in coordinating protein complexes at the plasma membrane during infection. The second largest network (II; n = 78) associated with lesion area is enriched in GO categories traditionally associated with the innate immune response in *Arabidopsis* (S7 Table). Despite the observation that natural variation in the jasmonate or salicylate hormone receptor was not associated with lesion size, lesion network II contains a large portion of genes with putative annotations related to defense and includes two of the major hubs from camalexin network II *ZAR1* (At3g50950; degree = 4) and the uncharacterized SAM-dependent methyltransferase (At1g55450; degree = 4). Lesion network III was enriched in GO categories related to autophagy and catabolism and included the large hubs that are known for being involved with amino acid and lipid metabolism (ASP4; At1g62800 and 2OG; At3g50210) (Fig 4B; S7 Table). The next largest network (IV) is also linked to catabolic and metabolic processes as it is enriched in GO categories linked to catabolism and one of the largest hubs is homogentisate 1,2-dioxygenase, a well-studied enzyme in the tyrosine catabolic pathway (*HGO*; At5g54080, degree = 7) (Fig 4B; S7 Table). Thus, these two networks suggest that variation in catabolic processes is linked to plant defense. Lastly, lesion network V is largely comprised of unknown and uncharacterized genes, including the two largest hubs within the network (At1g10020, At4g03110). Including the unknown genes, this network is enriched for GO categories related to trehalose and polysaccharide metabolism and includes genes like SUGAR_TRANSPORTER_1 (STP1; At1g11260) and CELLULOSE_SYNTHASE_LIKE_C4 (CSLC4; At3g28180), which suggests that this network is related to polysaccharide metabolism and callose wall deposition. Thus, these co-expression networks illustrate the broad array of mechanisms that may be variable and influence quantitative resistance to *Botrytis cinerea* in *Arabidopsis thaliana* and they provide new gene targets and testable hypotheses for future experiments.

### Validation of Associated Gene Involvement from Mapping

**Fig 6:**
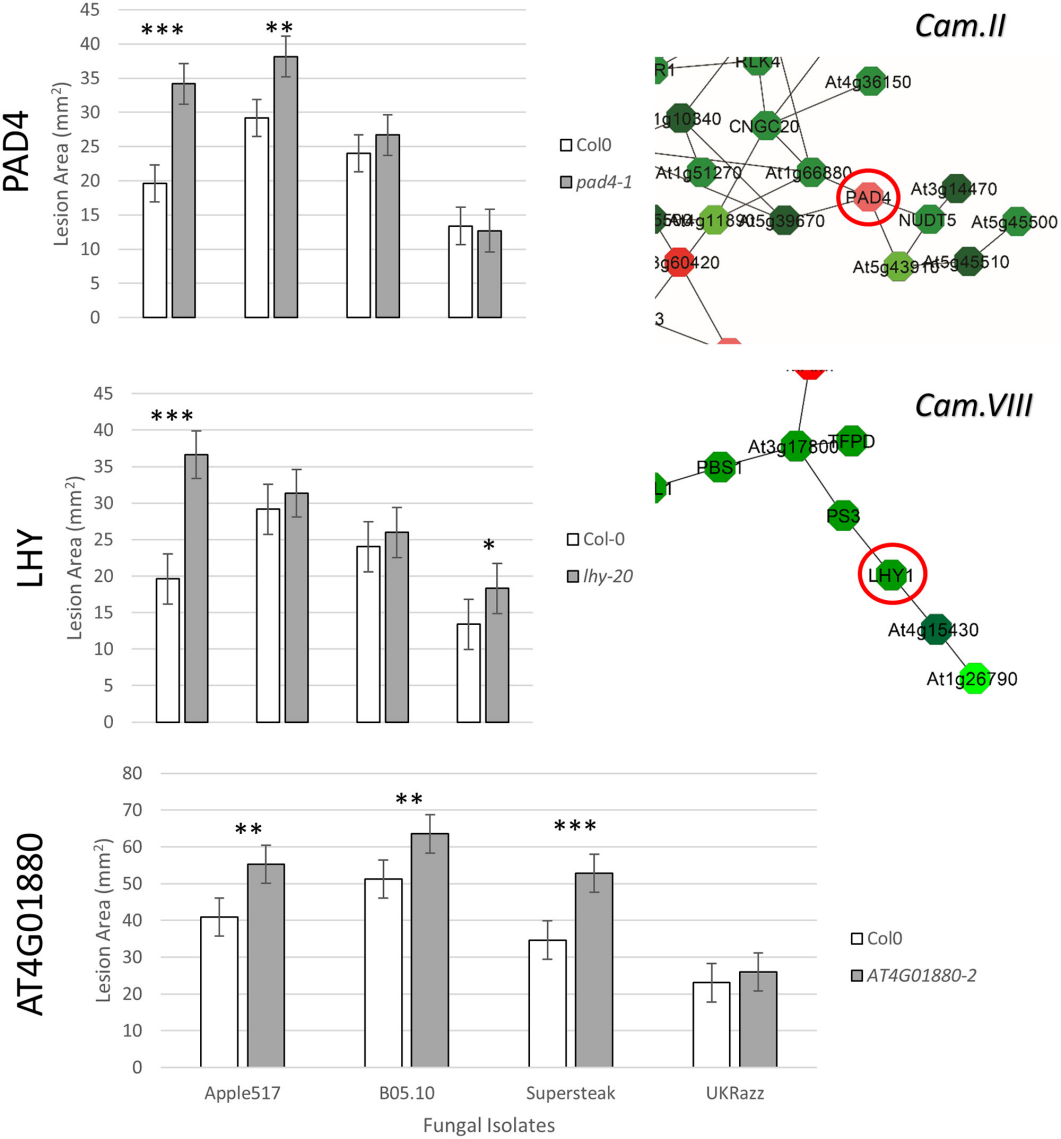
Lesion area validation. Bargraphs showing the model corrected means for the lesion area on wildtype Col-0 versus *pad4-1*, *lhy-20*, and *at4g01880-*2 mutants. Relevant co-expression networks for PAD4 and LHY1 are shown to right of the bargraph. Significance of the difference in camalexin accumulation between Col-0 and the mutant with each isolate are shown as P< 0.001 ‘***’, 0.001 ‘**’ and 0.01 ‘*’.

Given the modular construction of the co-expression networks and the common concern with false positives within GWA data, we selected several genes that were present within co-expression networks and a set of genes that were not within co-expression networks to validate their involvement in quantitative resistance to *B*. *cinerea*. All of these genes were required to have at least two SNPs crossing the 0.95 threshold as previously published [43]. Specifically, we selected a total of 22 genes associated with either camalexin production or lesion area whose homozygous and viable mutants could readily be obtained (S9 Table). In addition, to test if the lack of GWA hits on known candidate genes was potentially caused by our selection of isolates, we selected three genes previously identified to be associated with quantitative resistance to *B*. *cinerea*, but were not found in either association studies, including PAD3 [65], TGA3, and ANAC0155 [55]. Individual gene knockouts in a Col-0 background were obtained from both the *Arabidopsis* Biological Resource Center, as well as several other laboratories that regularly work with these mutants, and each line was tested for homozygosity. Similar to the GWA experiments, each mutant and a wildtype Col-0 control was grown in a randomized complete block design with three experimental replicates and four independent biological replicates. At five weeks of growth, the first five mature leaves of each plant was removed and infected with either one of the previous isolates (Apple517, B05.10, Supersteak, UKRazz) or a mock inoculation. Infected leaves were imaged at 72 hpi and collected for camalexin measurement via HPLC. Pictures were analyzed using the same image analysis pipeline described above for the GWAS. Of the 24 genotypes planted, we were unable to obtain validated and viable homozygous seed for seven mutant lines (S9 Table). Mutant genotypes for seven of the 15 remaining genes tested demonstrated significant differences for camalexin production in comparison to wildtype, and mutant alleles in six of the 15 genes showed altered disease resistance as measured by lesion area (Tables 2 and S10–S11) (Figs 5 and 6). Of the six lines that altered lesion development, five of them had increased lesion size in the knockout lines while the CESA2 mutant actually showed increased resistance to *B*. *cinerea* in comparison to WT (S10 Table). As such, we can identify both susceptibility and resistance genes via this approach. Interestingly, there was less evidence of isolate specificity within the single gene analysis than we found using the accessions, which could indicate that the isolate specificity is more environmentally variable as suggested by the three-way interaction of environment with isolate and plant genotype (Tables 2 and S11). Alternatively, this could imply that the highly polygenic nature of resistance among the accessions complicates the ability to compare measures isolate specificity between individual genotypes and accessions. However, it should be noted that including multiple isolates to test every gene did increase our level of replication and likely benefited our power to detect a phenotypic difference above what would have happened with a single isolate.

**Table 2:** Significance of plant genotype terms for validated genetic lines. Summary of p-values of an F-test from a GLM comparing each mutant separately to wildtype Col-0 using type II sums of squares. The GLM includes single terms for the experiment replicate, isolate genotype, and plant genotype as well as all two-way and three-way interaction terms. An expanded table can be found in Supporting Data.

					Camalexin (ng/mm)		Lesion Area (mm^2^)	
AGI	Gene Name	Network	Cam. Assoc.	Lesion Assoc.	Plant	Plant:Isol	Plant	Plant:Isol
AT4G14400	ACD6-3	Cam.II/Les.XV	Yes	Yes	0.8862	0.5673	0.1579	0.7901
AT4G17010	-	-	Yes	Yes	0.3076	0.3693	0.3583	0.7860
AT2G39940	COI1-1	Cam.I	Yes	-	<0.0001	0.0001	< 0.0001	0.0911
AT3G52430	PAD4-1	Cam.II	Yes	-	0.0921	0.3138	0.0018	0.0836
AT2G25540	CESA10-1	Cam.V	Yes	-	0.0218	0.5020	0.0029	0.4393
AT1G01060	LHY-20	Cam.VIII	Yes	-	0.0213	0.2094	0.0071	0.0910
AT1G14690	MAP65-7	-	Yes	-	0.4819	0.3428	0.6053	0.7947
AT2G32150	-	-	Yes	-	0.6152	0.2091	0.7059	0.8165
AT4G01860	-	-	Yes	-	0.0006	0.3303	0.8072	0.5901
AT4G01880	AT4G01880-1	-	Yes	-	0.0435	0.2783	0.1930	0.6387
AT4G01880	AT4G01880-2	-	Yes	-	0.0334	0.3325	0.0048	0.6017
AT4G01883	-	-	Yes	-	0.0052	0.1182	0.1501	0.6259
AT4G14368	-	-	Yes	-	0.7912	0.5391	0.5008	0.7703
AT4G14420	AT4G14420	Les.I	-	Yes	0.0028	0.6789	0.0529	0.9669
AT1G31260	ZIP10-1	-	-	Yes	0.8411	0.2348	0.15250	0.4136
AT4G39350	CESA2-1	-	-	Yes	0.6023	0.6765	0.0257	0.3726
AT1G22070	TGA3-2	-	-	-	0.5514	0.4955	0.4908	0.6121
AT3G15500	ANAC055-1	-	-	-	0.2106	0.3892	0.4111	0.3127
AT3G26830	PAD3-1	-	-	-	<0.0001	0.0004	<0.0001	0.0143

To assess the impact of significance thresholds on our validation efforts, we compared our validation candidates with the presence of SNPs at different significance thresholds. There were no SNPs in any of the candidate genes at a 0.999 significance threshold indicating that this threshold would have a high false-negative error rate with this set of genes, which would result in missing the nine genes that did validate. Of the 15 genes tested, six had one or more SNPs at the 0.99 threshold and these genes validated at a slightly higher rate than the genes with just two or more SNPs at the 0.95 threshold (67% v 56% when using all traits) (S12 Table). Interestingly, there was no difference in the validation rate between genes with a 0.99 threshold SNP that were within networks or absent from these networks (67% in both cases) (S12 Table). While this suggests that most of the increase in validation rate was achieved by requiring two or more SNPS, there were only three genes in these two groups and more tests would be required to assess if the network approach helped identify true positives. While the genes with a 0.99 threshold validated at a slightly higher level than genes with two or more SNPs at the 0.95 threshold, the fact that 56% of the genes with the two SNPs at the lower threshold also validated shows that the 0.99 threshold still imparts a high false-negative error rate. Thus, this test provides some empirical insight into how to set a significance threshold for calling candidate genes to attempt and validate depending upon the level of false positive and false negatives that a researcher is willing to accommodate in their experimental design.

## Discussion

The host innate immune response is extremely complex in nature involving a myriad of biological processes resulting in disease incidence and severity that is often quantitative, especially with regard to resistance to common endemic pathogens. However, it is unclear exactly how many genes are involved in quantitative resistance to endemic pathogens and which biological processes are most important. This study has drastically expanded the number of genes implicated with respect to quantitative *Botrytis*-resistance in *A*. *thaliana* and has illustrated the system level complexity inherent in host resistance. Further, while this study identified several known biological processes involved in plant immunity, such as vesicle trafficking and gene regulation, this study has also implicated new processes that have not previously been associated with *Botrytis*-resistance, including genes involved with development, protein turnover, and catabolism. Using GWA allowed us to dissect and validate a number of new genes even though the effects were relatively modest and the associated genes have significant interactions between the host genotype, pathogen genotype, and the environment. This suggests that quantitative resistance in plants may be a highly polygenic and conditional genetic network.

### Quantitative Resistance: More than R-genes

One current theory of evolution of innate immunity is that it is primarily driven by the presence/absence of *R-*gene combinations, including RLKs and NLR proteins [2,3,66,67]. This would imply that natural variation in quantitative resistance is merely the differential ability of host genotypes to directly detect the presence of a pathogen via PAMPs, or to indirectly detect the impact of pathogen effectors, and trigger the appropriate downstream response. Next generation genomic re-sequencing efforts have difficulty in resolving genetic variation at resistance gene clusters, which could potentially cause us to underestimate the contribution of *R*-genes to natural variation in quantitative resistance [68–71]. However, the effect of polymorphisms at known *R*-gene clusters is not drastically larger than other loci identified (S13 and S14 Figs; S12 Table) and thus would not lead to a significant alteration in the resulting conclusion. While we did see enrichment in the putative receptor RLKs and NBS-LRR proteins in genes associated with quantitative resistance, they only account for a small proportion of the genes implicated in quantitative variation in the innate immune response. Interestingly, we observed a much stronger enrichment in cytoplasmic localized NLR proteins relative to the plasma membrane bound RLKs. Taken with the fact that *Botrytis cinerea* does not have a type III secretion system or form haustoria, this implies that resistance to *B*. *cinerea* in *Arabidopsis* is primarily driven by indirect pathogen detection by monitoring of host cellular components. Therefore, it appears that it may be more efficient for the plant host to monitor the health of its own cellular components rather than attempting to directly detect the myriad potential pathogen PAMPs and effectors to determine its infection state. This may also help explain why genes were implicated in an isolate specific manner as different genotypes of the pathogen may have differential effects on host specific processes. In addition, this may also help explain why endophytic microbes that are phylogenetically related to plant pathogens do not elicit an immune response in the host.

### The Power of GWA to Understand Complex Phenotypes

In contrast to studies looking at quantitative resistance in defined breeding populations [32–34,72,73], we were able to identify a vastly larger pool of candidate genes that could be validated with a 60% success rate (Table 2). This GWA method was likely more successful than previous efforts to map quantitative resistance loci for multiple reasons related to the specific experimental design and statistical methods used. For instance, by removing a large portion of the large genetic outliers and major population structure from the host mapping population, we were left with a core set of natural *Arabidopsis* accessions that share a large fraction of common allelic variants and have reduced cryptic population structure. In addition, the use of the ridge regression mapping method allows for testing all polymorphisms within the population in a single model by treating them as random variables. This obviates the need for post-hoc multiple testing corrections that can arbitrarily increase false negatives. However, this makes it difficult to assign significance and p-values for SNP-association since it is difficult to assign accurate degrees of freedom for each variable. To resolve this issue, we used a permuted a significance threshold to identify heteroscedastic SNP effects that are larger than can be expected by a random association. This method identified thousands of moderate effect SNPs associated with a change in quantitative resistance relative to the population mean, which likely represent an upper bounds on the number of causal loci for this population. These are more loci than have been identified in previous studies, and thus necessitates developing methods to filter results and test for false positive associations. To address this, we reduced the number of loci into gene co-expression networks derived from all publically available microarrays in Arabidopsis [64]. These smaller collections of genes are both genetically associated with *Botrytis* resistance and are typically expressed at the same time, in the same tissues, and under the same treatments. This implies that genes within a network function together during the transition to an immune response during pathogen attack. However, it should be noted that both genetic mapping and co-expression analyses have known issues with false-positive associations due to the underlying assumptions in each method. For instance, genetic linkage can falsely implicate a gene that is directly adjacent to a causal gene in genetic mapping analyses and gene co-expression is often insufficient to imply co-function. However, since these limitations represent different and independent assumptions, we leveraged the two methods against each other to filter out false positives within each method and obtain a more detailed biological interpretation of the results. Thus, we were able to achieve a much more accurate picture of quantitative resistance by selecting a population with reduced structure using appropriate statistical approaches, and leveraging complementing datasets.

### The Effect of the Pathogen in the Interaction and Isolate Specificity

Importantly, our study shows that the genetic background of the fungal isolate plays a significant role in identification of plant disease resistance genes. While this has long been shown in presence/absence of functional alleles in several biotrophic pathogens, the importance of the pathogen genotype for quantitative, necrotrophic interactions is only recently being rediscovered [74]. Here, we attempted to use the genetic and phenotypic diversity in *B*. *cinerea* to challenge the innate immune system of *Arabidopsis* in as wide a range as possible. This enhanced our ability to detect genes associated with quantitative resistance as the most of gene networks are made up of genes identified as candidates for resistance to individual isolates. In addition, we were able to further enhance our ability to identify genes associated with quantitative resistance by mapping multiple phenotypic descriptions of infection. For example, the uncharacterized putative methyltransferase At4g01880 was only identified by mapping for camalexin associated loci in response to the B05.10 isolate. However, a T-DNA knockout of At4g01880 significantly influenced both camalexin and lesion area for multiple isolates upon single gene validation. These findings argue for analysis of multiple phenotypes and isolates to differentially probe the host innate immune system. Further, since there was very little overlap between the genes identified among the isolates, this argues that our estimates of the genes identified in this study are a more conservative estimate and that we have not identified all genes associated with quantitative resistance.

Lastly, this isolate specificity has potential long term implications concerning the design and development of durable resistance for quantitative pathogenic interactions. The isolate specificity in genes associated with quantitative resistance seem to imply that there is no single network common to resistance among genetically distinct isolates of the pathogen. However, individual pathogen genotypes appear to affect different loci of genes that are commonly co-expressed. This implies that each isolate is a core set of genes associated with quantitative resistance. However, these components can only be discovered through the use of multiple, phenotypically distinct pathogenic isolates.

### Conclusion

Overall, quantitative resistance appears to be dependent on the efficient functioning of several cellular processes and must be able to deal with a number of potential inputs. This study has generated several new broad hypotheses to explore, such as what is the impact of developmental genes on quantitative resistance and are the core components of quantitative resistance functionally canalized? The large list of candidate genes we identified in this study as altering quantitative resistance provides the raw genetic material to begin asking numerous functional questions with regard to quantitative resistance including 1) how does a host interpret the biological inputs to understand that it is infected, 2) how does a host weight those inputs relative to other environmental and physiological inputs, 3) how does a host chose to respond to a particular pathogen and is the response canalized for all similar pathogens, 4) is the host or the pathogen the larger determining factor in the interaction. By developing approaches to study these putative quantitative resistance networks in combination and in isolation, we can begin to understand how this intricate system functions across diverse pathogens and environments. In addition, a better understanding of quantitative resistance should help breeding efforts to for improved disease resistance.

## Methods

### Population Selection and Growing Conditions

A collection of 96 natural accessions of *Arabidopsis thaliana* were selected from a larger population of 241 accessions based on similar flowering time (63.1 ± 0.95 (s.e.) days to flowering) [42]. Using accessions with similar flowering time limits the potential effects of ontogenic variation of relative plant age across our population while, for an unknown reason, also removing extreme genetic outliers (Fig 1 and S1 Fig). Seeds of each accession were cold stratified in 0.1% Phytagar for 4 days at 4°C in the dark. Three seeds per accession were sown on soil (Sunshine Mix #1, Sun Gro Horticulture, Agawam, MA) randomly in 104-cell flats and were covered with a clear humidity dome. Seeds were allowed to germinate and seedlings were thinned to one plant per cell at one week of age. Seedlings were allowed to mature for 5 weeks at 22°C with 10 hours of full spectrum light. A total of four flats were planted and the experiment was repeated two weeks after the initial sowing, which allowed for a total of 8 independent biological measurements per accession in a 4-by-2 randomized complete block design. Plants were watered bi-weekly as needed.

At five weeks of growth, the first five mature leaves were collected for infection and placed on growing flats lined with 1% Phytagar. The spores of five-day-old sporulating cultures of four phenotypically and genotypically diverse *Botrytis cinerea* isolates (Apple517, B05.10, Supersteak, UKRazz) [50,51] on peach slices were collected in 1/2x grape juice, counted using a hemacytometer, and diluted to 10 spores/μL. Detached leaves were infected with 4 μL of the spore solution, which allows for approximately 40 spores per leaf, and leaves from each plant were randomly assigned either a 1/2x grape juice control or one of the isolate spore dilutions as previously described [33,34,75]. This level of inoculation allows for lesion progression that is sufficiently moderate to optimize the ability to quantify the infections progression over time. Infected leaves were incubated at room temperature for 72 hours, at which point pictures were taken of the expanding lesion using an 18 Mp high resolution T3i Canon camera outfitted with an EF-S 10-22mm f/3.5–4.5 USM ultra-wide angle lens, and infected leaves were collected for chemical analysis. Pictures of developing lesions were taken every 24 hours at a final resolution of approximately 10 pixels per mm.

### Automated Image Analysis

Lesion areas were measured using a semi-automated image analysis script using the open-source R statistical environment [76] enabled with the EBImage and CRImage packages [52,77]. Leaf objects were automatically identified as green, highly saturated objects in the image and lesions were identified as brown, low saturation object in the leaf. Image masks were generated for both the leaf and lesion, which were manual refined by a technician to ensure proper object calling. The area of these leaves and lesions were then automatically measured in the number of pixels per lesion and converted to area using a 1 cm reference contained within each image.

### Camalexin Measuring

Camalexin was extracted and measured as previously described [75]. Briefly, the leaves were ground in 400μL of 90% methanol, centrifuged to remove precipitates, and the supernatant was transferred to a fresh plate. A total of 50 μl of the extract was run on an Agilent Lichrocart 250–4 RP18e 5 μm column using an Agilent 1100 series HPLC. Camalexin was detected using a DiodeArray (DAD) at 330 nm and with a fluorescence detector at emission 318 nm/excitation 385 nm. Separation was achieved using the following program with aqueous acetonitrile: 5 min gradient from 63% to 69% acetonitrile, 30 sec gradient from 69% to 99% acetonitrile, 2 min at 99% acetonitrile and a post-run equilibration of 3.5 min at 63% acetonitrile [75]. Purified camalexin was used to produce a standard curve to identify and quantitate camalexin.

### Statistics and Association Mapping

To select *Arabidopsis* accessions for the GWA population, publically available SNP data was collected from the *Arabidopsis* 1,001 genomes project [40,70,78–80] for all accession available on campus. The EMMA package in R [46] was used to generate the kinship matrix for all related accessions. Accessions were filtered based on common flowering time [42] (63.1 ± 0.95 (s.e.) days to flowering), which also removed the most extreme genetic outliers. Relative genetic groups were qualitatively determined.

Experimental and treatment effects on each phenotype were determined using a multivariate linear model. To explore the experimental effects on our phenotypes, we used one of two linear models:

$${Cam}_{etaip}\;=\;\beta 0\;+\;E_{e}\;+\;T_{t}\;+\;A_{a}\;+\;{T(I)}_{ti}\;+\;{A(P)}_{ap}\;+\;{A:T}_{at}\;+\;{A:T(I)}_{ati}\;+\;{T:P}_{tp}\;+\varepsilon_{etaip}$$

$${Lesion}_{eiap}\;=\;\beta 0\;+\;E_{e}\;+\;I_{i}\;+\;A_{a}\;+\;{A(P)}_{ap}+\;{A:I}_{ai}+\;\varepsilon_{eiap}eiap$$

The main fixed effects are denoted as E, T, A, I, and P, which represents the experimental block, infection treatment, accession background, isolate background, and plant respectively. In addition, e = 1,2; t = 1,2; a = 1,…,95; i = 1,…,5; and p = 1,…,400. The residual error was assumed to be normal (ɛ ~ *N*(0,σ^2^)). Infection treatment was not included in the lesion model as no lesions were observed on mock treated tissue and control tissue was not included in the dataset. Both models showed a highly significant interaction between the accession genetic background and the isolate genetics background (Table 1), as such, all downstream analysis was conducted within an isolate background. Broad-sense heritability (*H*^2^) and genetic grouping was calculated as a proportion of the explained variance for each isolate using the following equation:

$$y_{efga}\;=\;\beta 0\;+\;E_{e}\;+\;{F(E)}_{ef}\;+\;G_{g}\;+\;{A(G)}_{ga}\;+\;+\;\varepsilon_{efga}$$

where the main effects are denoted as G, A, E, and F to represent group, accession, experiment block, and flat block respectively, and g = 1,…, 5; e = 1,2;f = 1, …,4; and a = 1,…,95. Again, residual error was assumed to be normal (ε_gaef_ ~ *N*(0, σ_ε_^2^)). To look for a relationship between camalexin production and lesion area, we used the non-parametric Spearman’s rank correlation test using model corrected means for each *Arabidopsis* accession within each isolate treatment.

Since very little variance for each phenotype can be attributed to the genotypic structure of the population, we opted for a ridge regression approach [54]. This approach models the effects of all polymorphisms in a single model by treating them as random effects, which avoids the use of post-hoc multiple testing procedures. From this model, the heteroscedastic effects (HEM) were extracted for each polymorphism in lieu of SNP-based best linear unbiased predictors in accordance with Shen, 2013 [54]. Since determining the degrees of freedom for random variables is difficult, and thus corresponding p-values, a significant effect threshold was determined by randomly permuting the phenotypic means across the accession backgrounds 1,000 times and taking the 99^th^ quartile. Individual genes were called associated with the phenotype if they had at least 2 significant SNPs in their coding region.

### Network Analysis

*A*. *thaliana* genes associated with quantitative resistance to any of the four *B*. *cinerea* isolates were condensed into co-expression networks determined using the ATTEDII database, as described by Chan, 2011. Briefly, Pearson’s correlation coefficients and mutual ranks (MR) were collected for all pairs of significantly associated genes in the database. Interactions were filtered by MR rank < 15. Putative function of the top 5 largest networks was qualitatively determined by the function of the well-studied genes in their membership. Several candidate genes from many of the larger networks were selected for downstream validation.

### Unbiased and R-gene Unbiased GO Enrichment Determination

Gene ontology (GO) enrichment for biological processes within the large 5 networks for each phenotype was determined using the Bioconductor packages org.At.tair.db, topGO, and goProfiles in the R statistical environment. The unbiased approach compared all associated genes against all genes that could potentially be called associated (at least two SNPs with MAF > 0.2) within this population. *R-*gene enrichment was determined using a hypergeometric test comparing associated RLKs [58] and NBS-LRR [59] proteins with all *R-*genes that could be called in the background of the unbiased approach.

### Gene Validation

Single gene T-DNA knockout lines in a Col-0 background were obtained and genotyped to be homozygous using standard PCR-based indel markers developed from the SALK Institute Genomic Analysis Laboratory (http://signal.salk.edu/tdnaprimers.2.html). Plants were grown, infected, and phenotyped for lesion size and camalexin against all four *B*. *cinerea* isolates and a mock control as described above. Plant were sown in a complete block design with three experimental replicates and four biological replicates in each block, thus providing 12 measurements per *A*. *thaliana* mutant / *B*. *cinerea* isolate combination. An F-test was conducted for each T-DNA line relative to Col-0 using one of the following GLMs:

$${Cam}_{etgi}\;=\;\beta 0\;+\;E_{e}\;+\;T_{t}\;+\;G_{g}\;+{\;T(I)}_{ti}\;+\;{G:T}_{gt}\;+\;{G:T(I)}_{gti}\;+\varepsilon_{etgi}$$

$${Lesion}_{eig}\;=\;\beta 0\;+\;E_{e}\;+\;I_{i}\;+\;G_{g}\;+\;{G:I}_{gi}+\;\varepsilon_{eig}$$

where the main fixed effects are denoted as E, T, G, and I, which represents the experimental block, infection treatment, genetic background, and isolate background respectively, with e = 1,2; t = 1,2; g = 1,2; and i = 1,…,5. The residual error was assumed to be normal (ɛ ~ *N*(0,σ^2^)).

## Supporting Information

S1 Fig Kinship matrix for available *Arabidopsis* accessions. Dendrogram of the EMMA kinship matrix for all available *Arabidopsis thaliana* accessions available at the start of the project. The mapping collection of 96 accessions were pulled from this population of 238 natural accessions.
(TIF)

S2 Fig Geographical map of *A*. *thaliana* accessions. A map illustrating the geographical distribution of where the *Arabidopsis thaliana* accession were collected. Closed circles indicate an accession that was used for mapping while open circles indicate that the accession was not used. The color of the closed circles describe the genomic group from the phylogenic analysis as follows: red (I), brown (II), green (III), blue (IV), pink (V).
(PDF)

S3 Fig Violin plots for induced Camalexin production among the isolates and control. Violin plots illustrating the distribution of induced camalexin production among the isolates and the un-infected control.
(TIFF)

S4 Fig Violin plots for lesion area among the isolates. Violin plots illustrating the distribution of lesion area among the isolates.
(TIFF)

S5 Fig Heatmap of Spearman’s correlation for camalexin and lesion area phenotypes. Spearman’s rank correlation of model corrected means for camalexin and lesion area and camalexin content across the *A*. *thaliana* accessions in response to each specific isolate. Color of the interactions range from red (a highly positive correlation) to blue (highly negative correlation) and a lack of correlation is indicated by white.
(TIF)

S6 Fig Manhattan plot for camalexin production in mock treated leaves. Manhattan plot of the z-scaled, absolute value of the heteroscedastic SNP effects for basal camalexin accumulation within uninfected tissue. The alternating shades of color indicate the five chromosomes. The red line indicates the significance threshold of the 99^th^ quartile of the SNP effects from 1,000 permutations of the model.
(TIF)

S7 Fig Manhattan plots for camalexin and lesion area in Apple517 infected leaves. Manhattan plot of the z-scaled, absolute value of the heteroscedastic SNP effects for camalexin accumulation (blue) and lesion size (green) for tissue infected with *B*. *cinerea* isolate Apple517. The alternating shades of color indicate the five chromosomes. The red line indicates the significance threshold of the 99^th^ quartile of the SNP effects from 1,000 permutations of the model.
(TIF)

S8 Fig Manhattan plots for camalexin and lesion area in B05.10 infected leaves. Manhattan plot of the z-scaled, absolute value of the heteroscedastic SNP effects for camalexin accumulation (blue) and lesion size (green) for tissue infected with *B*. *cinerea* isolate B05.10. The alternating shades of color indicate the five chromosomes. The red line indicates the significance threshold of the 99^th^ quartile of the SNP effects from 1,000 permutations of the model.
(TIF)

S9 Fig Manhattan plots for camalexin and lesion area in Supersteak infected leaves. Manhattan plot of the z-scaled, absolute value of the heteroscedastic SNP effects for camalexin accumulation (blue) and lesion size (green) for tissue infected with *B*. *cinerea* isolate Supersteak. The alternating shades of color indicate the five chromosomes. The red line indicates the significance threshold of the 99^th^ quartile of the SNP effects from 1,000 permutations of the model.
(TIF)

S10 Fig Manhattan plots for camalexin and lesion area in UKRazz infected leaves. Manhattan plot of the z-scaled, absolute value of the heteroscedastic SNP effects for camalexin accumulation (blue) and lesion size (green) for tissue infected with *B*. *cinerea* isolate UKRazz. The alternating shades of color indicate the five chromosomes. The red line indicates the significance threshold of the 99^th^ quartile of the SNP effects from 1,000 permutations of the model.
(TIF)

S11 Fig Common genes at different permuted effects thresholds. A pdf of Venn diagrams illustrating the number of genes called in common among the isolates for both induce camalexin production and lesion area for a variety statistical thresholds (0.95, 0.975, 0.99, and 0.999).
(PDF)

S12 Fig Overlap of camalexin and lesion area associated host genes. Venn diagram illustrating the number of overlapping genes identified in both the camalexin and lesion area GWA mapping.
(TIF)

S13 Fig Common GO enriched categories for both camalexin and lesion area. Top gene ontogeny results that are in common between camalexin production and lesion area.
(TIF)

S14 Fig ATTEDII co-expression networks at different permuted effects thresholds. A pdf of co-expression networks of genes called at different permuted effects thresholds (0.95, 0.975, 0.99, and 0.999) from the GWA for induced camalexin production and lesion area.
(PDF)

S1 Table Summary of mapping results. Summary table describing the total number SNPs and genes tested and associated with each phenotype within the different *B*. *cinerea* isolate treatments.
(XLSX)

S2 Table Genomic group proportion of variance (η^2^) estimates. Estimates of the raw proportion of variance for the experiment relative to the proportion of *Arabidopsis* genomic group and accession nested in genomic group for each phenotype within each *B*. *cinerea* isolate treatment.
(XLSX)

S3 Table Associated gene tables. Tables of *Arabidopsis* genes with their chromosome and position associated and theie estimated effect from the ridge regression. Permuted thresholds for each phenotype are listed above.
(XLSX)

S4 Table Well-studied *Botrytis* resistance genes. A table of genes previously connected to *Botrytis* resistance according to Mengiste (2012). The table includes the number of SNPs per each gene with a MAF > 0.2 in the population and the number of SNPs associated with both camalexin production and lesion area. A secondary table is also included to identify exactly which SNPs in the population are associated with each phenotype.
(XLSX)

S5 Table *R-*gene enrichment. The table includes a list of putative *R-*genes, specifically annotated receptor-like kinases (RLKs; Lehti-Shiu et al., 2009) and Nod-like receptors (NLRs; Meyers et al., 2003b), associated camalexin and lesion area phenotypes, and calculated enrichment for associations.
(XLSX)

S6 Table Unbiased GO-enrichment. A table of all biological process GO-enrichment categories for all genes associated with induced camalexin production and lesion area relative to the total number genes tested by the ridge-regression.
(XLSX)

S7 Table Network GO-enrichment. Tables of biological process GO-enrichment categories for genes in the five largest co-expression networks for camalexin and lesion area relative to the total number genes tested by the ridge-regression.
(XLSX)

S8 Table Network traits. List of general network characteristics for the five largest co-expression networks associated with camalexin production and lesion area.
(XLSX)

S9 Table Gene validation summary. Summary of genes selected and tested for validation of involvement with *Botrytis*-resistance including a list of failed genetic lines.
(XLSX)

S10 Table Gene validation F-tests and LSMeans. F-tables of the camalexin and lesion area general linear models for each mutant line against wild-type Col-0 infected with each of the isolates. Model corrected means for each plant/isolate combination are also provided.
(XLSX)

S11 Table P-values of gene validation F-tests. A summary table of the p-values for each term in the camalexin and lesion area general linear models for each mutant against wildtype Col-0 from S12 Table.
(XLSX)

S12 Table Estimation of false positive and negative rates across the validation test set for different significance thresholds. False positives shows the fraction of mutant lines with no significant phenotype that had a GWA hit for that phenotype at the given threshold. False positives shows the fraction of mutant lines with a significant phenotype that had no GWA hit for that phenotype at the given threshold. Camalexin and Lesion show the trait for which there was a significant association given the threshold. The thresholds were threefold, the gene had two or more SNPs significant at the 0.95 threshold, had one SNP significant at the 0.99 threshold and was either in a coexpression network or not.
(XLSX)

S1 Dataset Camalexin networks. Cytoscape file containing co-expression networks associated with induced camalexin production.
(ZIP)

S2 Dataset Lesion area networks. Cytoscape file containing co-expression networks associated with lesion area.
(ZIP)
