# Supplementary figures and images for: The Quantitative Basis of the Arabidopsis Innate Immune System to Endemic Pathogens Depends on Pathogen Genetics
Source: PLoS Genet. 2016 Feb 11;12(2):e1005789. doi: 10.1371/journal.pgen.1005789 (PMC4750985; doi:10.1371/journal.pgen.1005789)

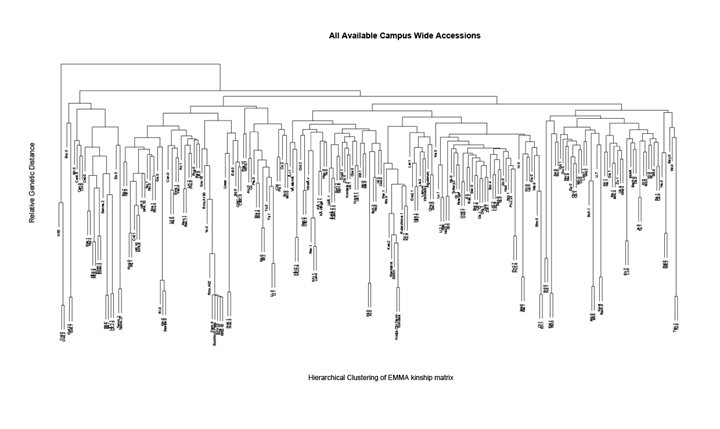

Supplement: S1 Fig — Dendrogram of the EMMA kinship matrix for all available Arabidopsis thaliana accessions available at the start of the project. The mapping collection of 96 accessions were pulled from this population of 238 natural accessions. (TIF) [file pgen.1005789.s001.TIF]

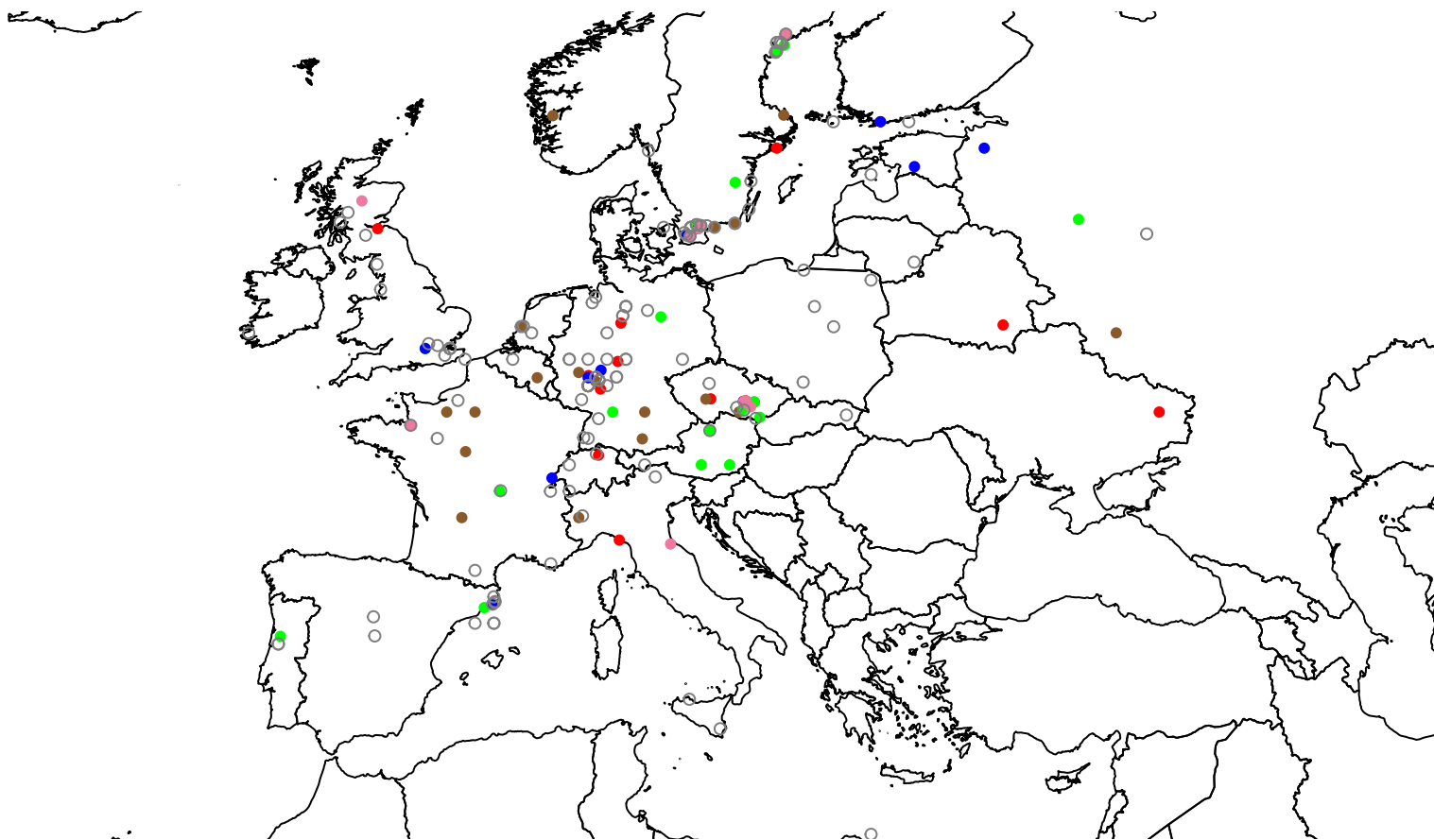

Europe

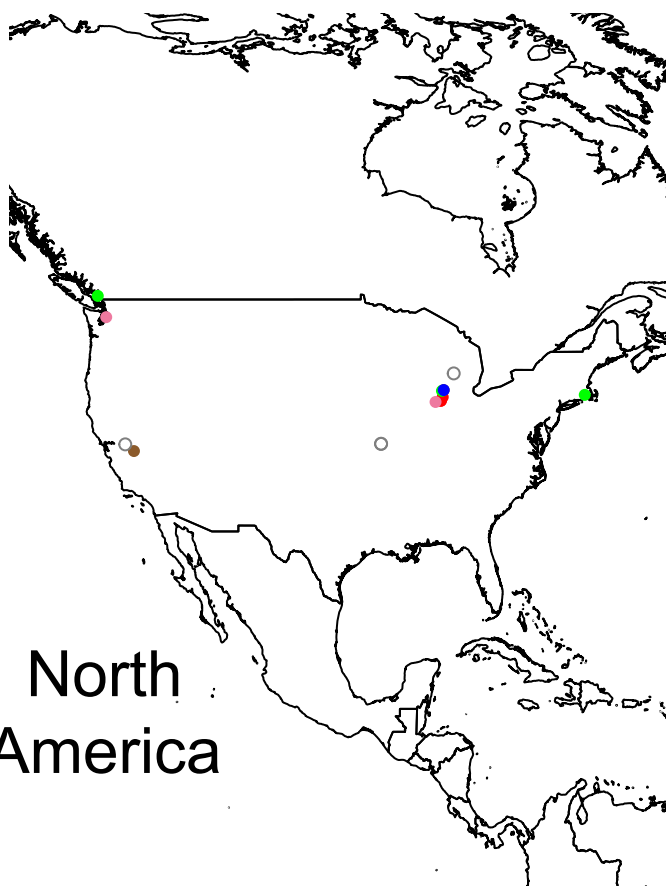

North  
America

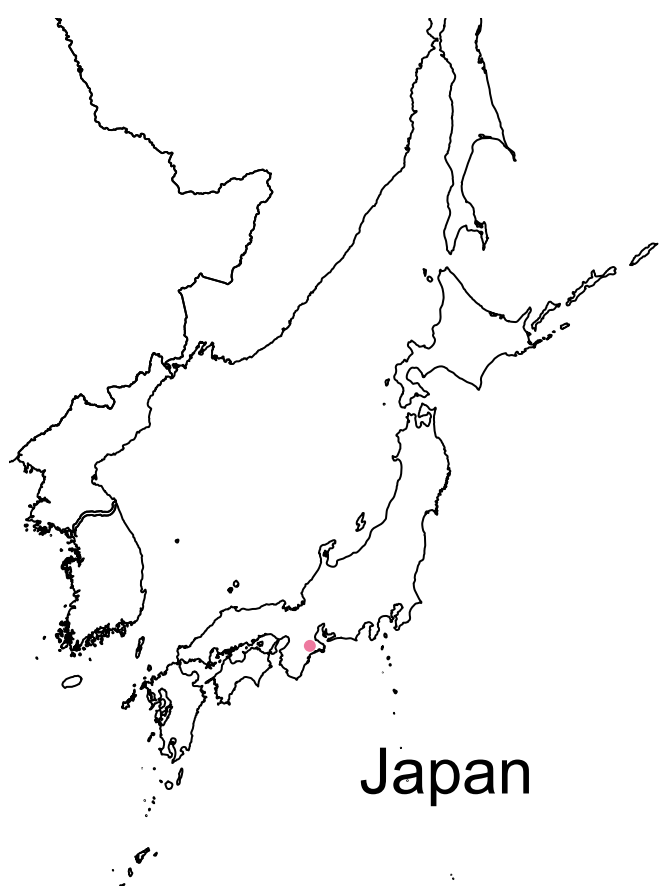

Japan

Supplement: S2 Fig — A map illustrating the geographical distribution of where the Arabidopsis thaliana accession were collected. Closed circles indicate an accession that was used for mapping while open circles indicate that the accession was not used. The color of the closed circles describe the genomic group from the phylogenic analysis as follows: red (I), brown (II), green (III), blue (IV), pink (V). (PDF) [file pgen.1005789.s002.pdf]

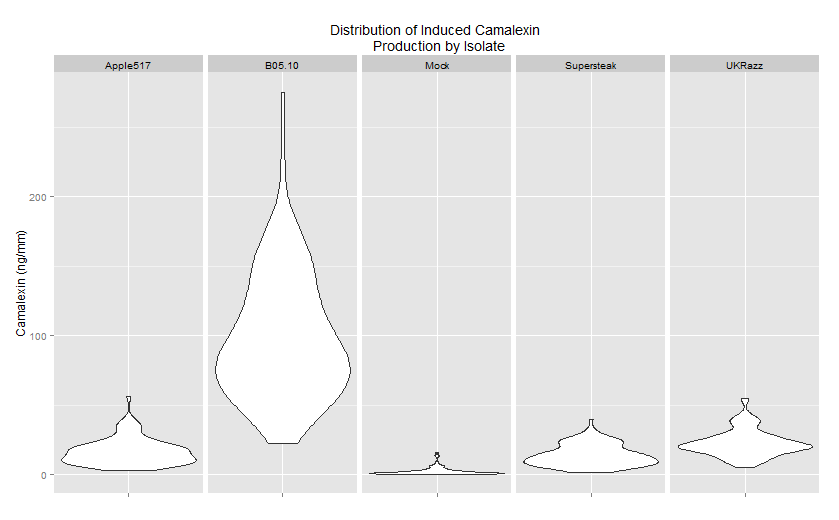

Supplement: S3 Fig — Violin plots illustrating the distribution of induced camalexin production among the isolates and the un-infected control. (TIFF) [file pgen.1005789.s003.tiff]

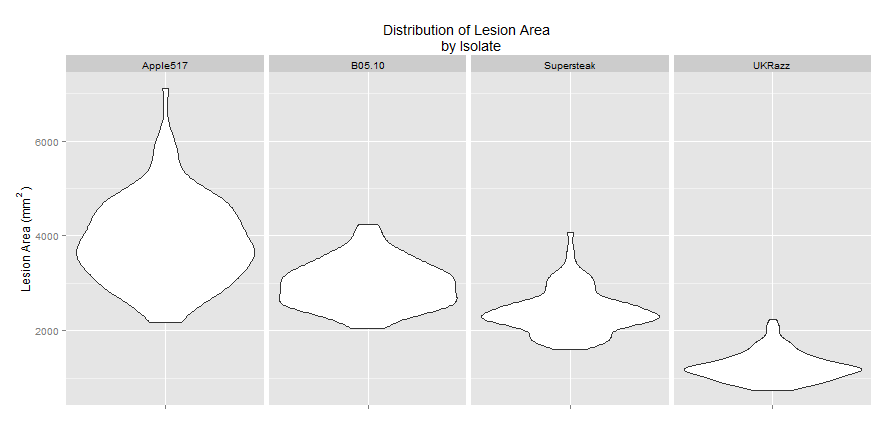

Supplement: S4 Fig — Violin plots illustrating the distribution of lesion area among the isolates. (TIFF) [file pgen.1005789.s004.tiff]

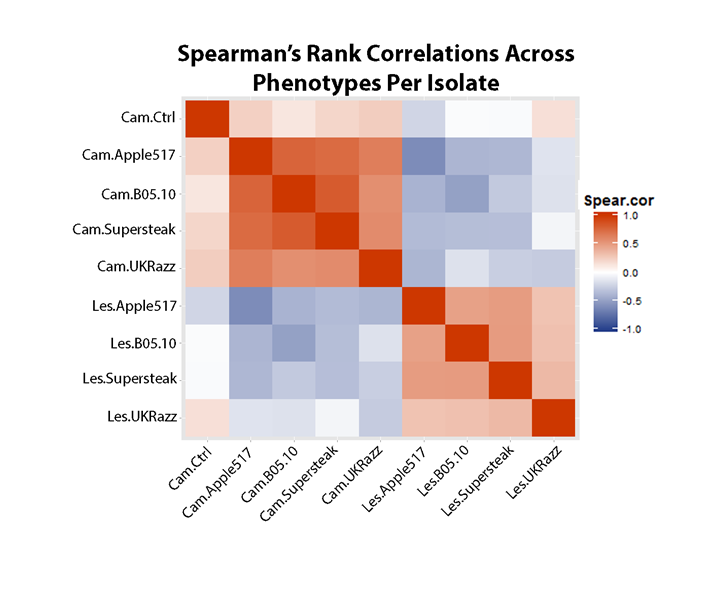

Supplement: S5 Fig — Spearman’s rank correlation of model corrected means for camalexin and lesion area and camalexin content across the A. thaliana accessions in response to each specific isolate. Color of the interactions range from red (a highly positive correlation) to blue (highly negative correlation) and a lack of correlation is indicated by white. (TIF) [file pgen.1005789.s005.TIF]

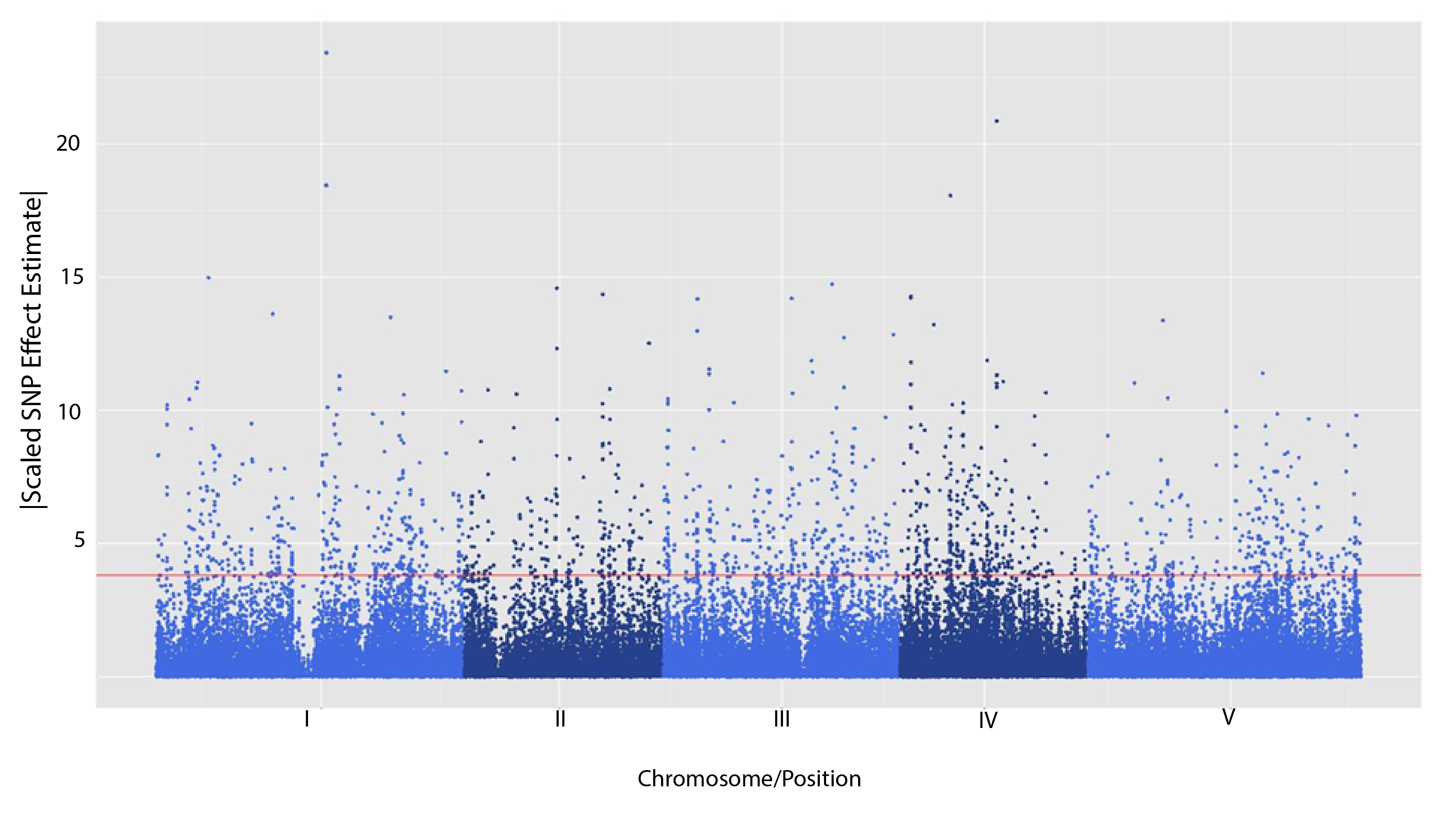

Supplement: S6 Fig — Manhattan plot of the z-scaled, absolute value of the heteroscedastic SNP effects for basal camalexin accumulation within uninfected tissue. The alternating shades of color indicate the five chromosomes. The red line indicates the significance threshold of the 99th quartile of the SNP effects from 1,000 permutations of the model. (TIF) [file pgen.1005789.s006.tif]

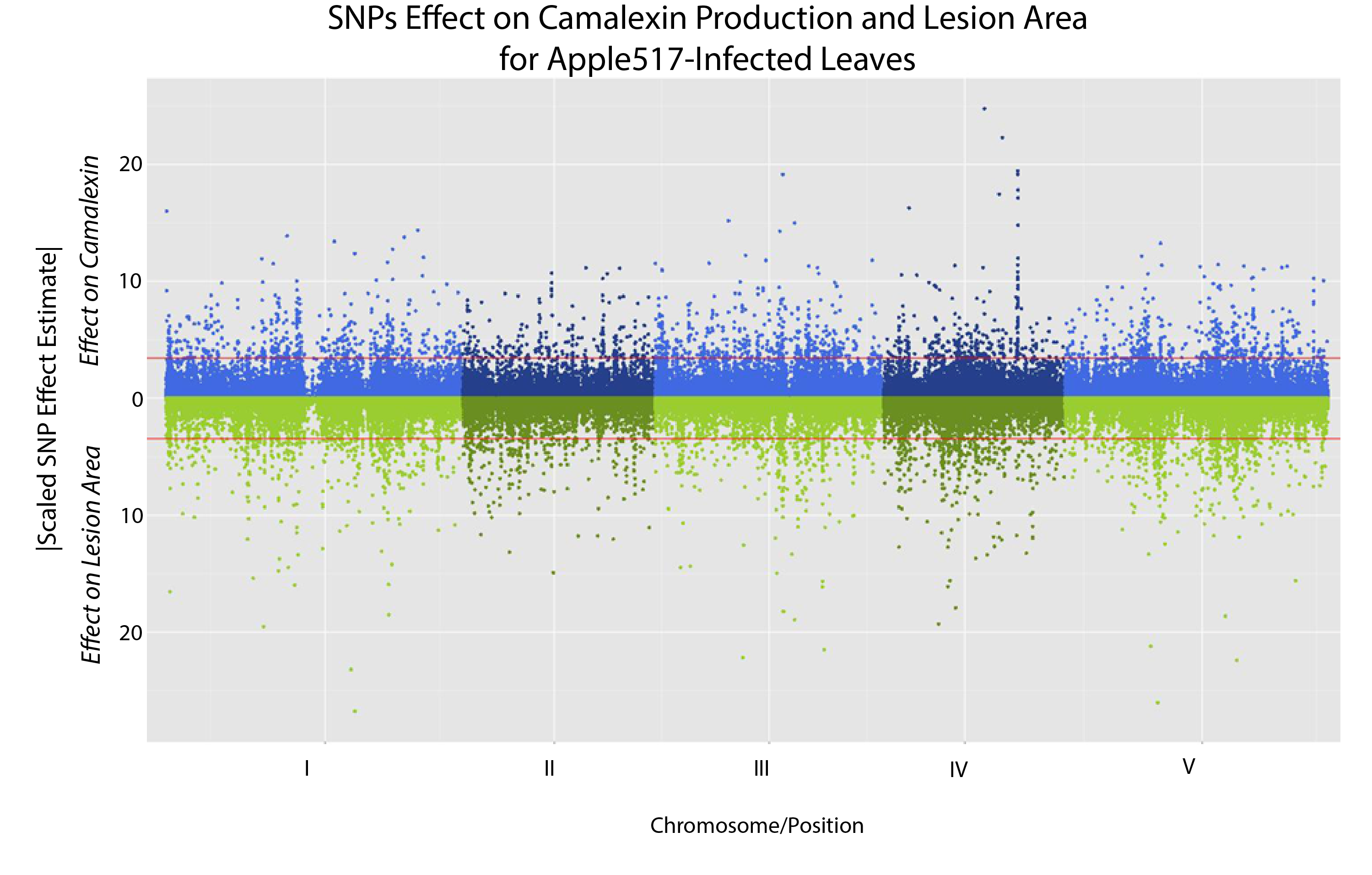

Supplement: S7 Fig — Manhattan plot of the z-scaled, absolute value of the heteroscedastic SNP effects for camalexin accumulation (blue) and lesion size (green) for tissue infected with B. cinerea isolate Apple517. The alternating shades of color indicate the five chromosomes. The red line indicates the significance threshold of the 99th quartile of the SNP effects from 1,000 permutations of the model. (TIF) [file pgen.1005789.s007.tif]

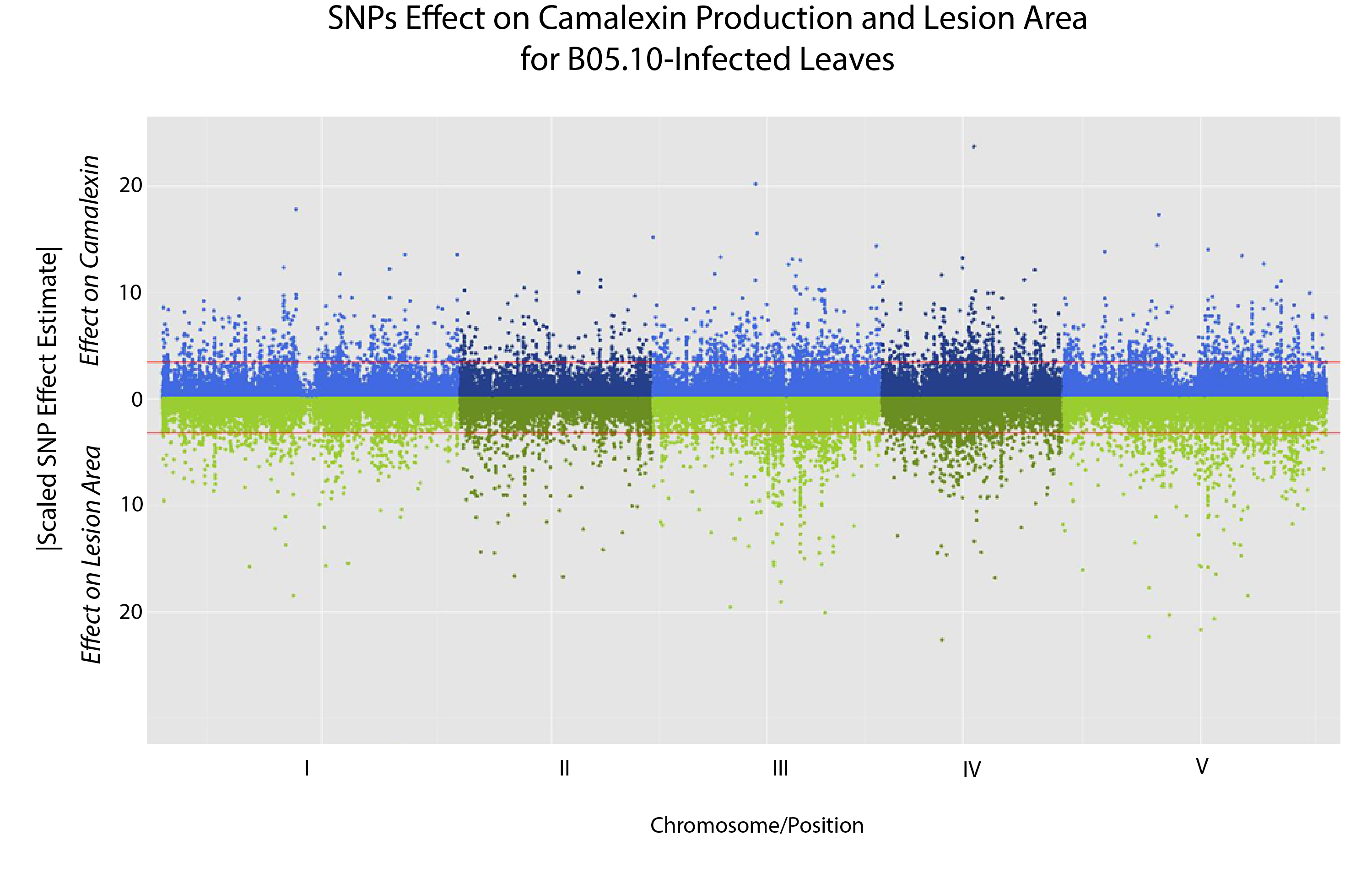

Supplement: S8 Fig — Manhattan plot of the z-scaled, absolute value of the heteroscedastic SNP effects for camalexin accumulation (blue) and lesion size (green) for tissue infected with B. cinerea isolate B05.10. The alternating shades of color indicate the five chromosomes. The red line indicates the significance threshold of the 99th quartile of the SNP effects from 1,000 permutations of the model. (TIF) [file pgen.1005789.s008.tif]

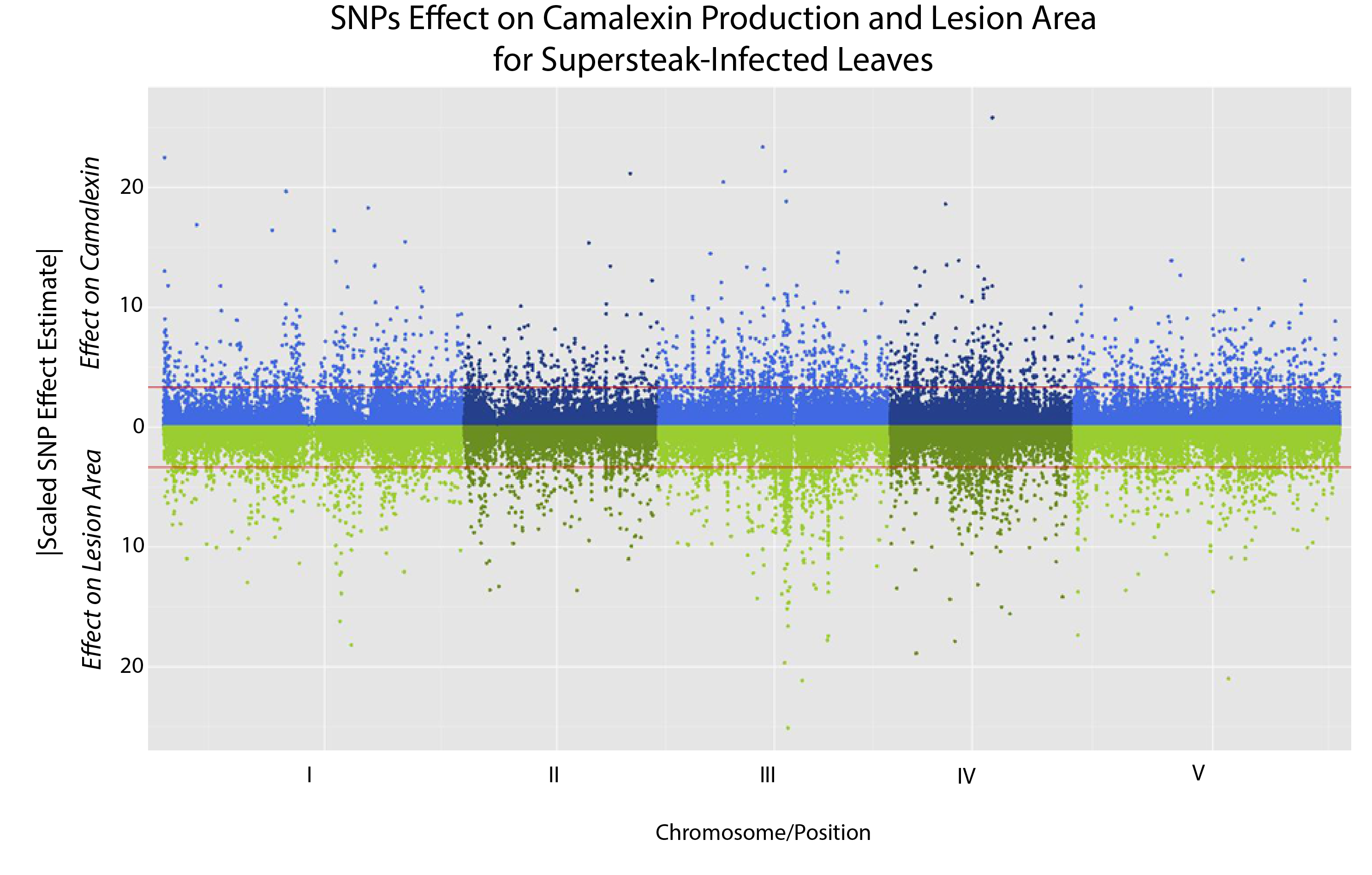

Supplement: S9 Fig — Manhattan plot of the z-scaled, absolute value of the heteroscedastic SNP effects for camalexin accumulation (blue) and lesion size (green) for tissue infected with B. cinerea isolate Supersteak. The alternating shades of color indicate the five chromosomes. The red line indicates the significance threshold of the 99th quartile of the SNP effects from 1,000 permutations of the model. (TIF) [file pgen.1005789.s009.tif]

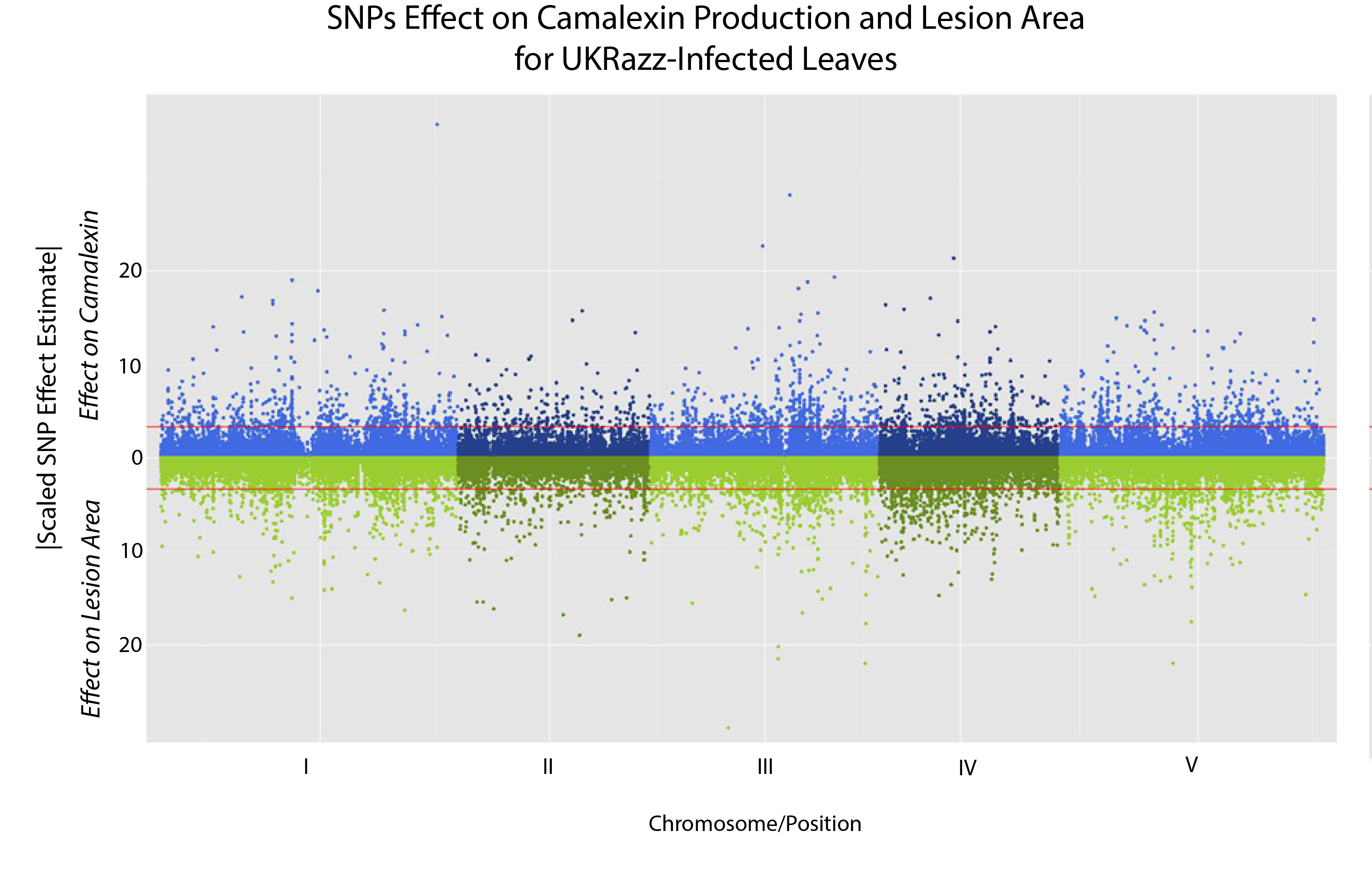

Supplement: S10 Fig — Manhattan plot of the z-scaled, absolute value of the heteroscedastic SNP effects for camalexin accumulation (blue) and lesion size (green) for tissue infected with B. cinerea isolate UKRazz. The alternating shades of color indicate the five chromosomes. The red line indicates the significance threshold of the 99th quartile of the SNP effects from 1,000 permutations of the model. (TIF) [file pgen.1005789.s010.tif]

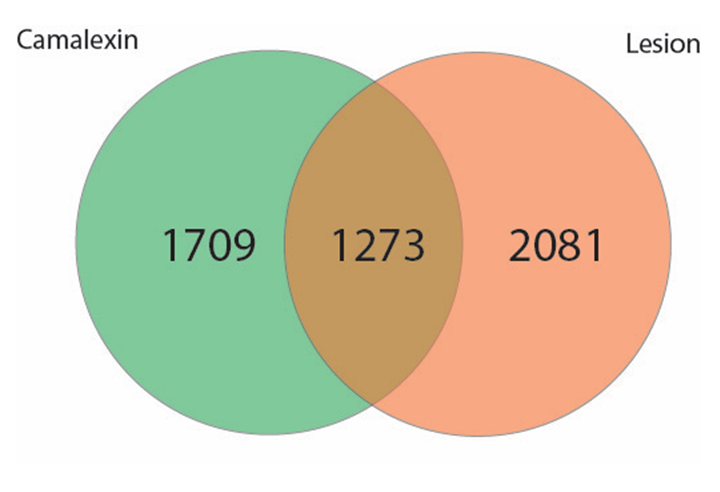

Supplement: S12 Fig — Venn diagram illustrating the number of overlapping genes identified in both the camalexin and lesion area GWA mapping. (TIF) [file pgen.1005789.s012.TIF]

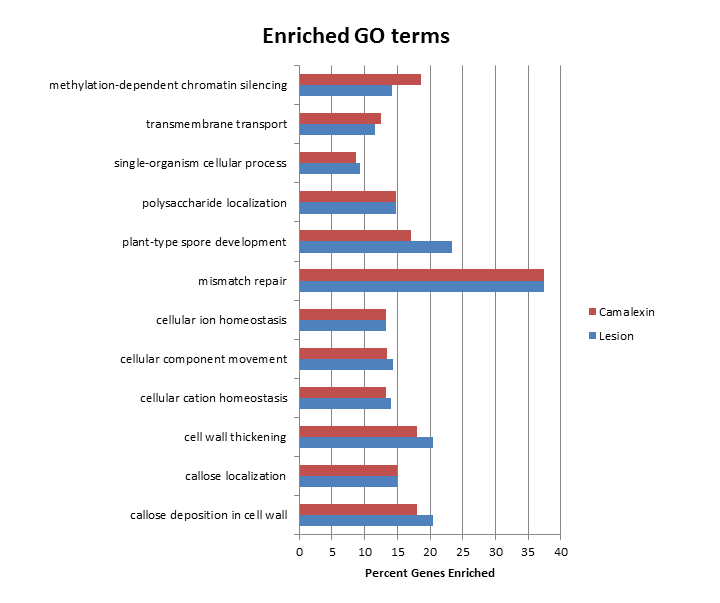

Supplement: S13 Fig — Top gene ontogeny results that are in common between camalexin production and lesion area. (TIF) [file pgen.1005789.s013.TIF]
