# Supplementary material for: The Quantitative Basis of the Arabidopsis Innate Immune System to Endemic Pathogens Depends on Pathogen Genetics
Source: PLoS Genet. 2016 Feb 11;12(2):e1005789. doi: 10.1371/journal.pgen.1005789 (PMC4750985; doi:10.1371/journal.pgen.1005789)
Supplement: S11 Fig — A pdf of Venn diagrams illustrating the number of genes called in common among the isolates for both induce camalexin production and lesion area for a variety statistical thresholds (0.95, 0.975, 0.99, and 0.999). (PDF) [file pgen.1005789.s011.pdf]

Summary Table of Genes Associated with Camalexin Production and Lesion Area of Botrytis-infected Leaves

	Camalexin (ng/mm)						*Lesion Area (mm^2)*			
Sig. Threshold	Apple515	B05.10	Ctrl	Supersteak	UKRazz		Apple515	B05.10	Supersteak	UKRazz
0.95	3693	3766	3394	3844	3845		3517	3794	3796	3666
0.975	1933	1937	1677	2040	2012		1767	1963	1968	1832
0.99	769	770	657	817	810		756	796	837	724
0.999	76	64	57	72	88		74	64	85	83

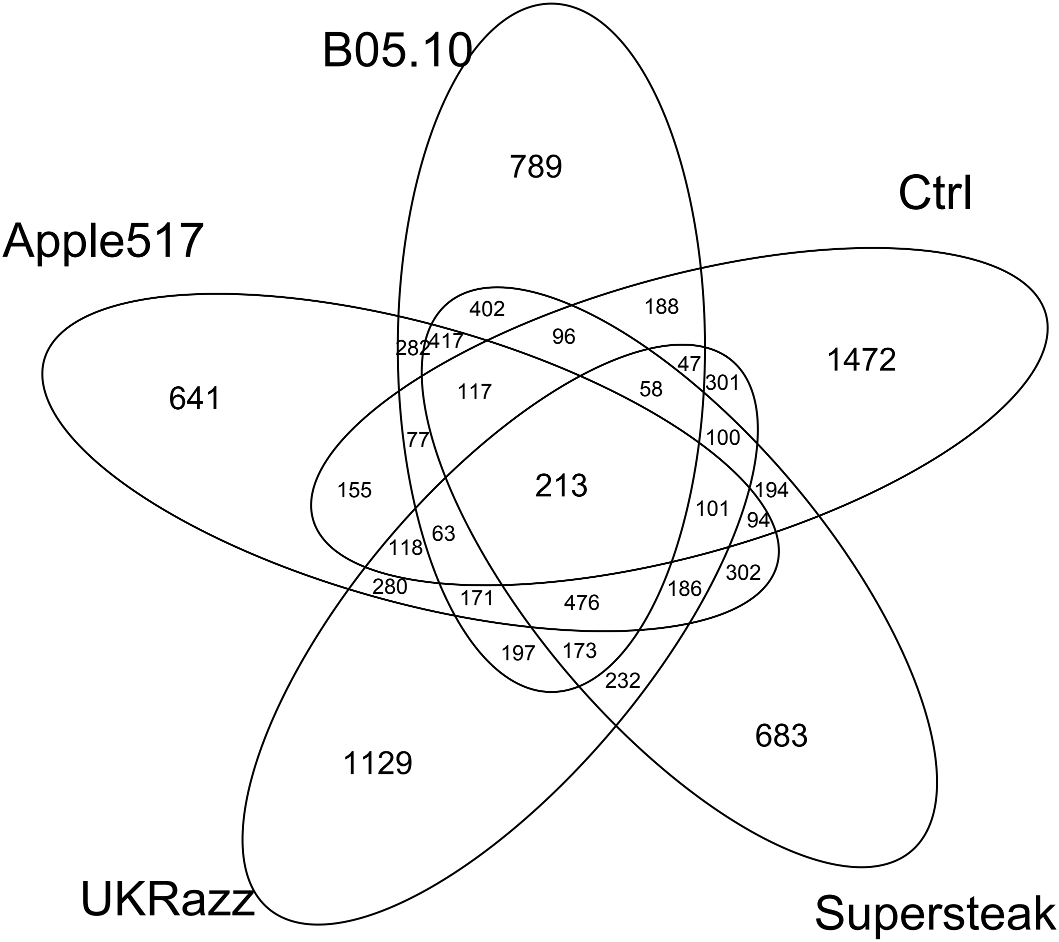

Overlap of genes called for camalexin production based on a 0.95 significance threshold.

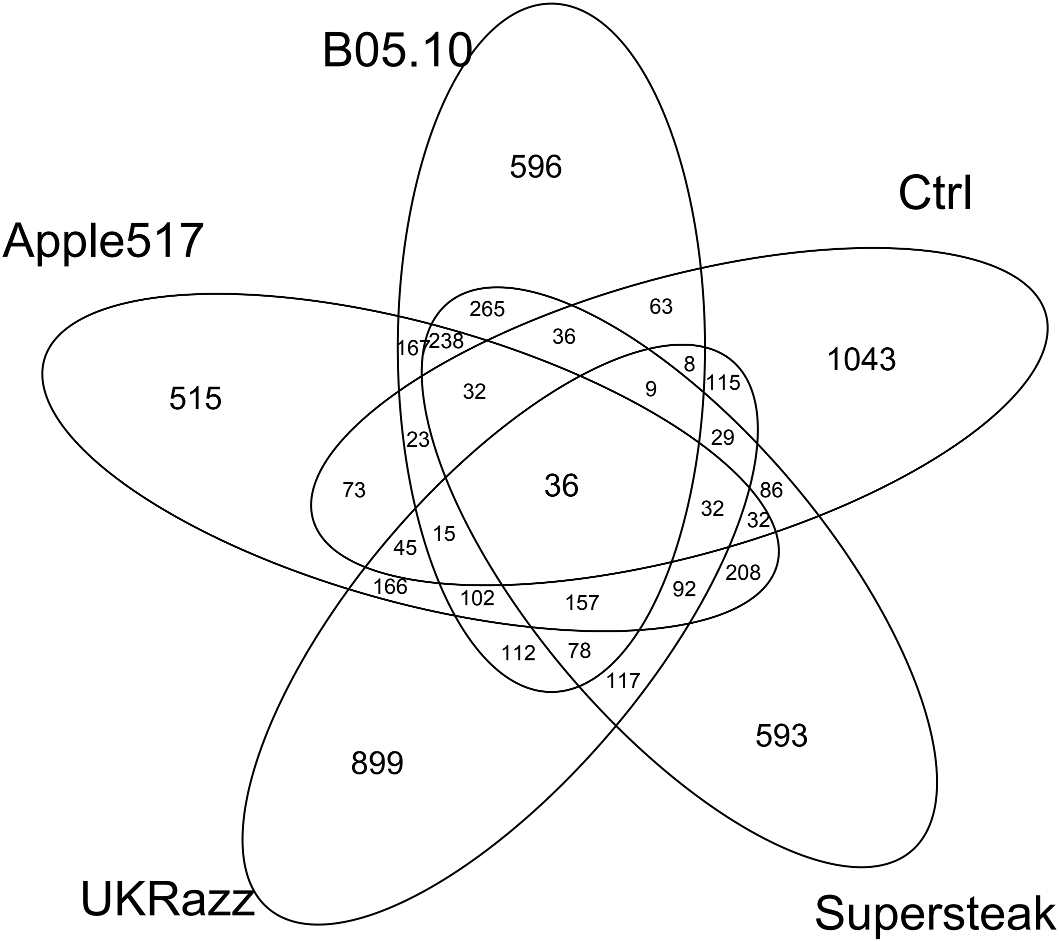

Overlap of genes called for camalexin production based on a 0.975 significance threshold.

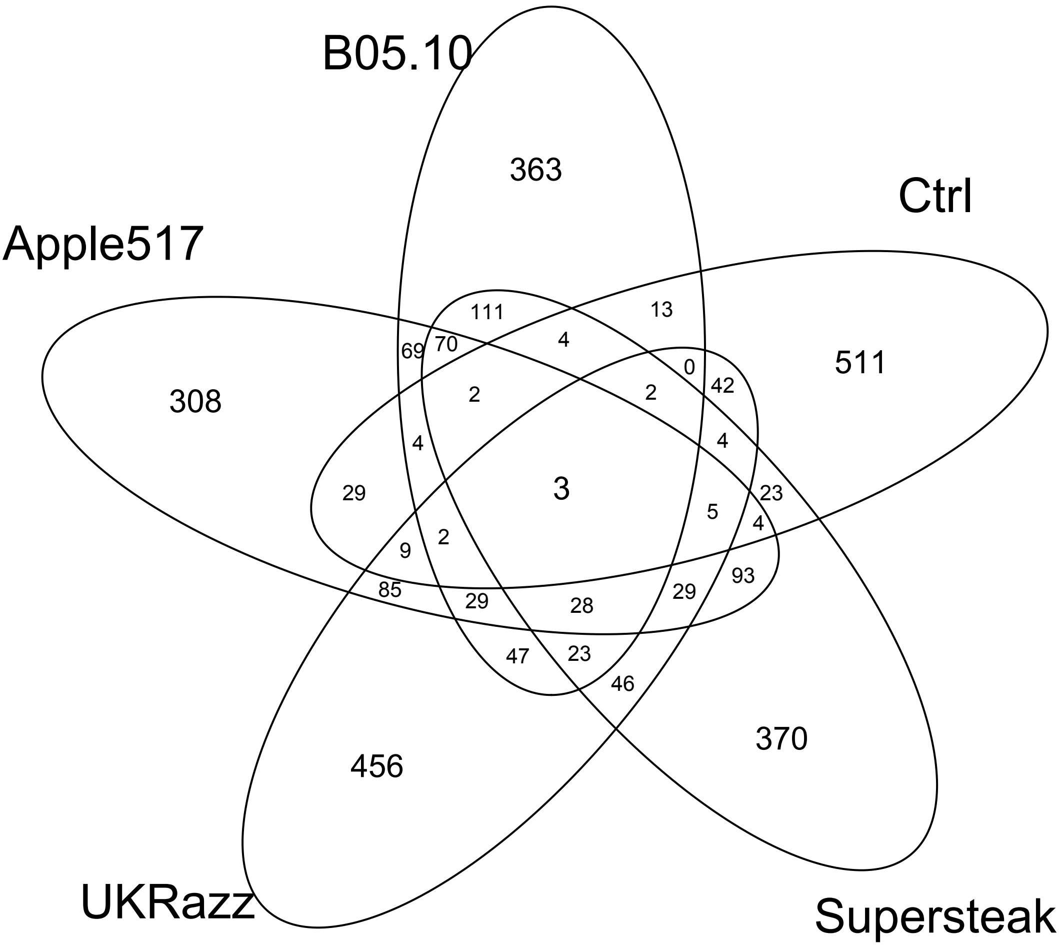

Overlap of genes called for camalexin production based on a 0.99 significance threshold.

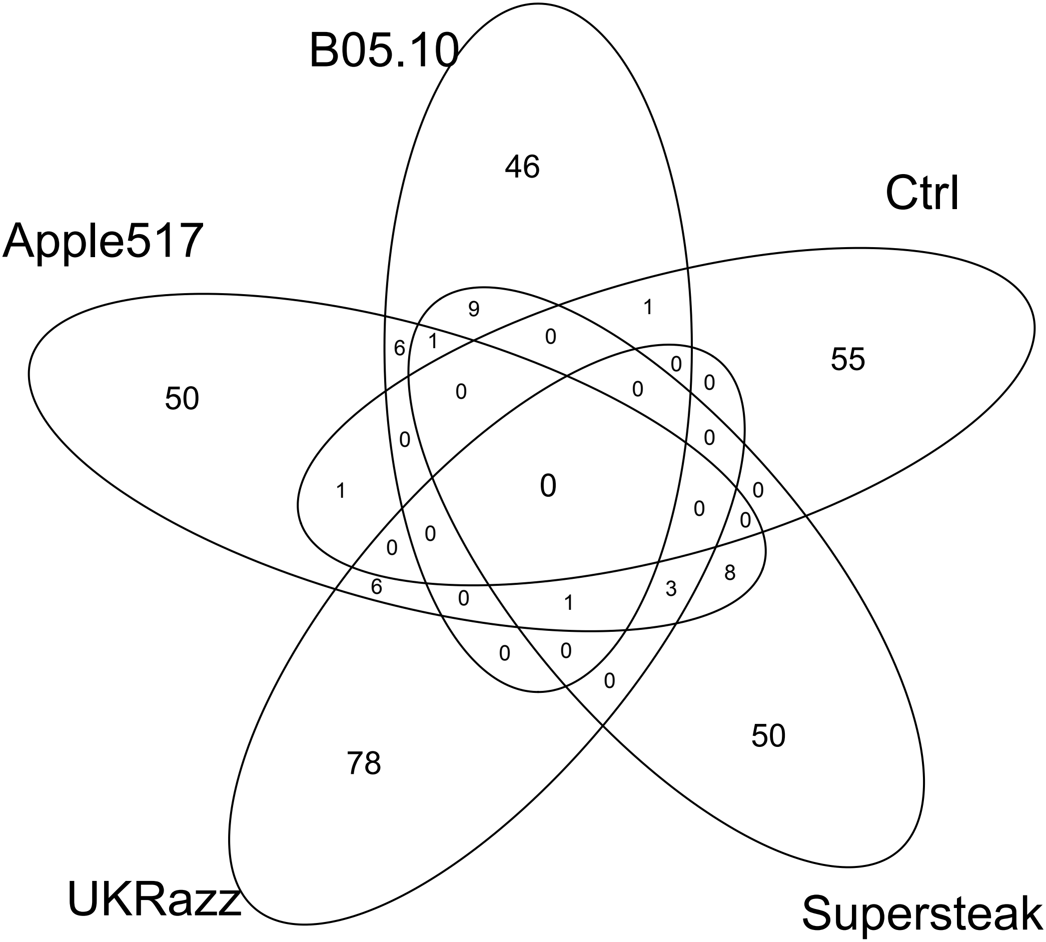

Overlap of genes called for camalexin production based on a 0.999 significance threshold.

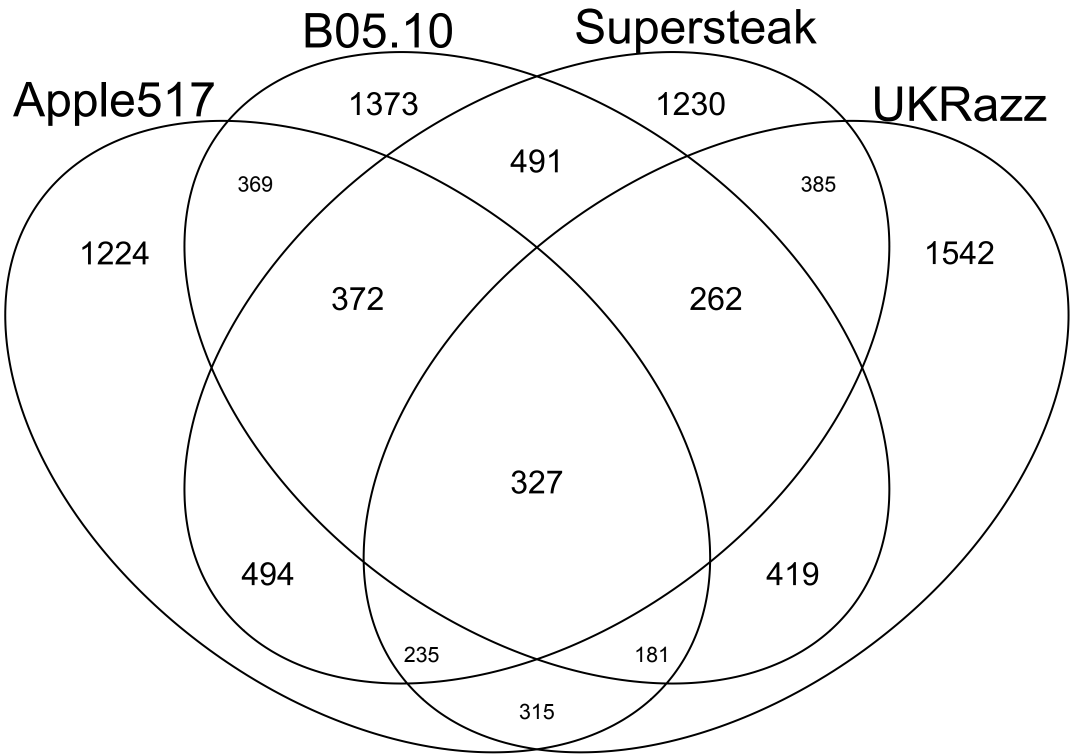

Overlap of genes called for lesion area based on a 0.95 significance threshold.

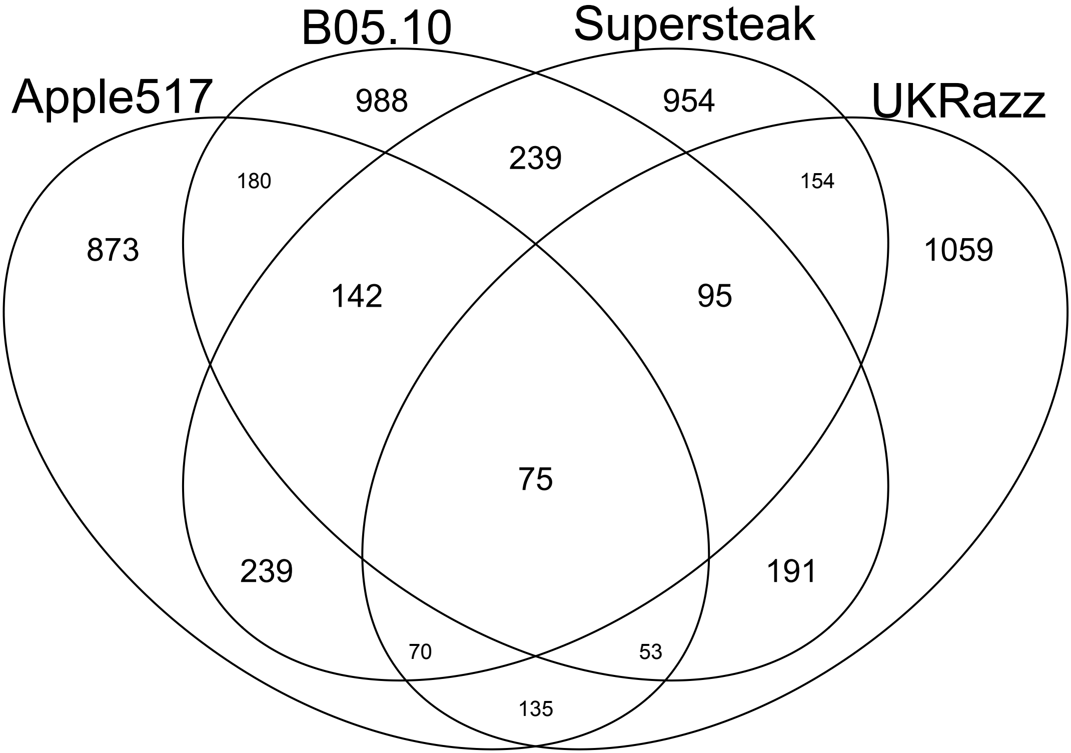

Overlap of genes called for lesion area based on a 0.975 significance threshold.

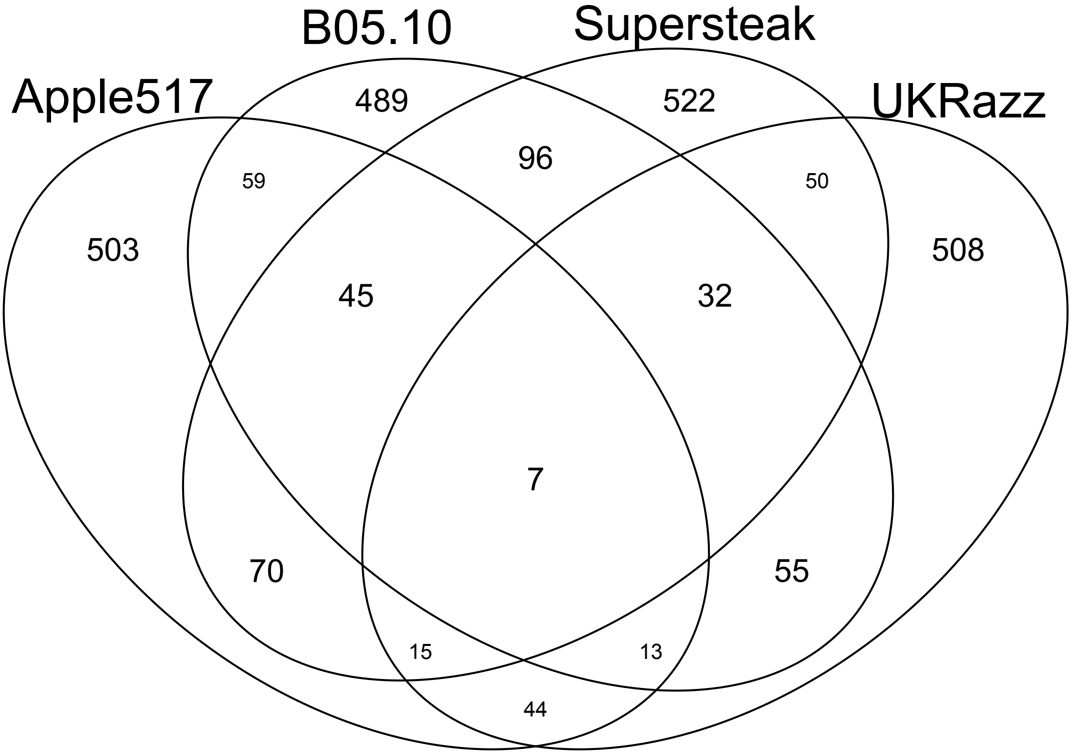

Overlap of genes called for lesion area based on a 0.99 significance threshold.

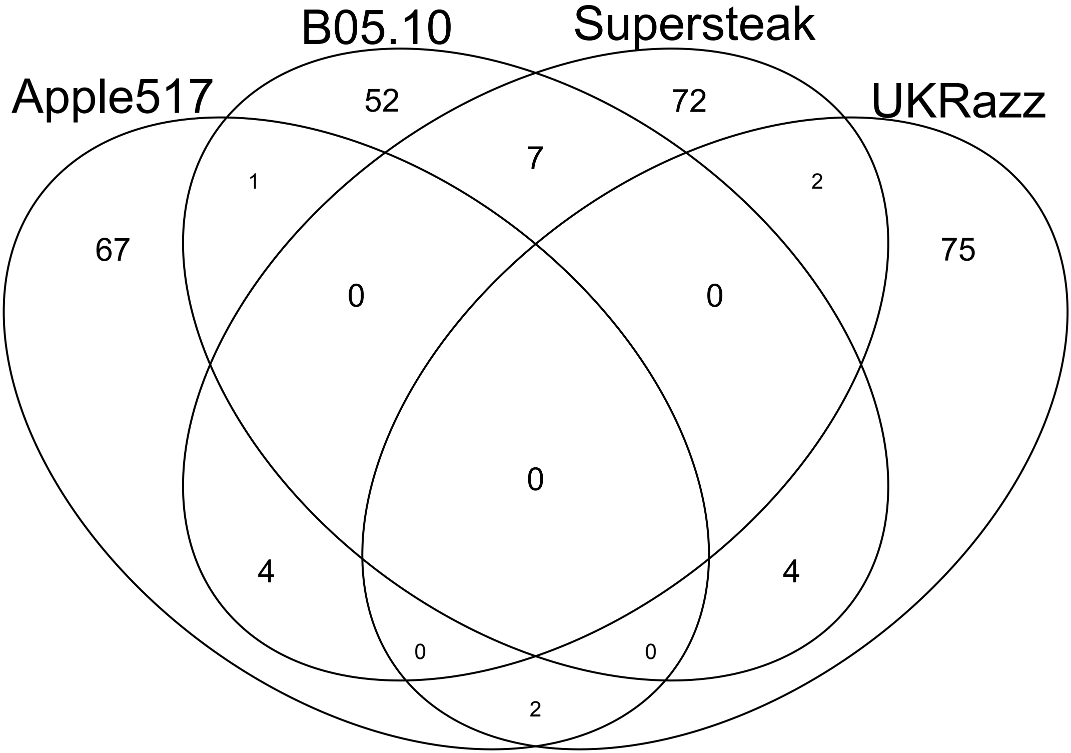

Overlap of genes called for lesion area based on a 0.999 significance threshold.
