# Supplementary material for: The Quantitative Basis of the Arabidopsis Innate Immune System to Endemic Pathogens Depends on Pathogen Genetics
Source: PLoS Genet. 2016 Feb 11;12(2):e1005789. doi: 10.1371/journal.pgen.1005789 (PMC4750985; doi:10.1371/journal.pgen.1005789)
Supplement: S14 Fig — A pdf of co-expression networks of genes called at different permuted effects thresholds (0.95, 0.975, 0.99, and 0.999) from the GWA for induced camalexin production and lesion area. (PDF) [file pgen.1005789.s014.pdf]

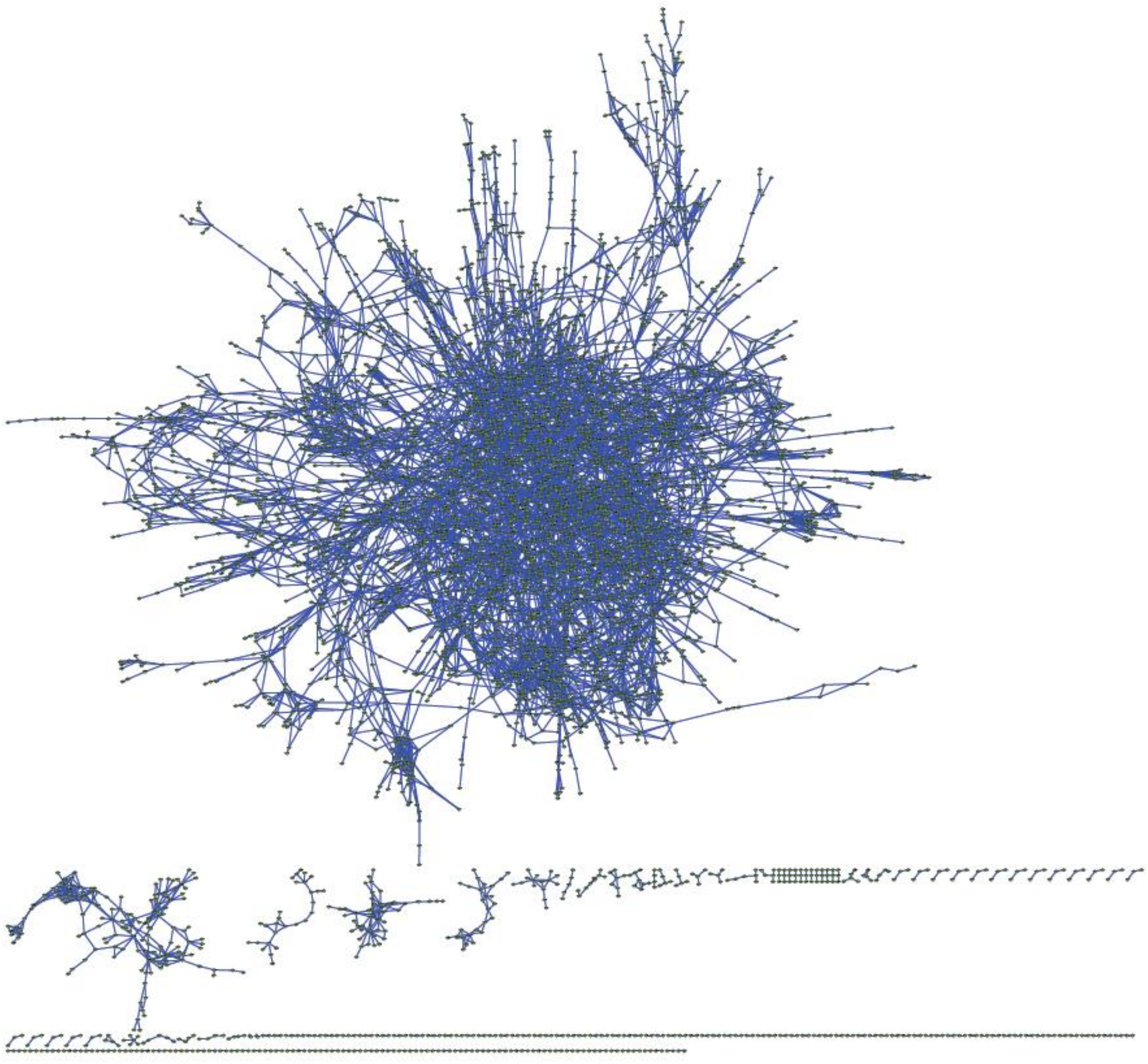

Camalexin Networks at a 0.95 threshold

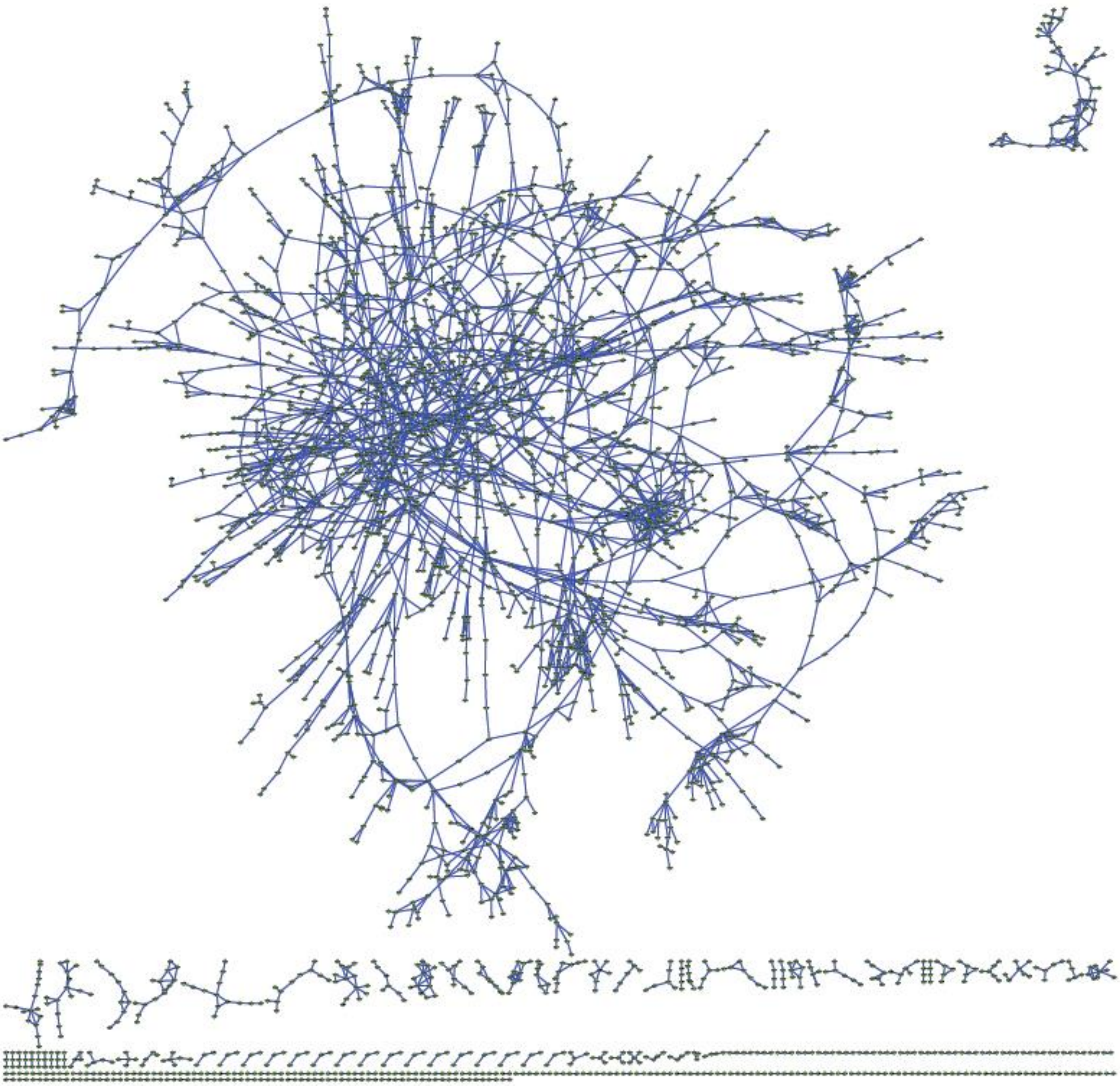

Camalexin Networks at a 0.975 threshold

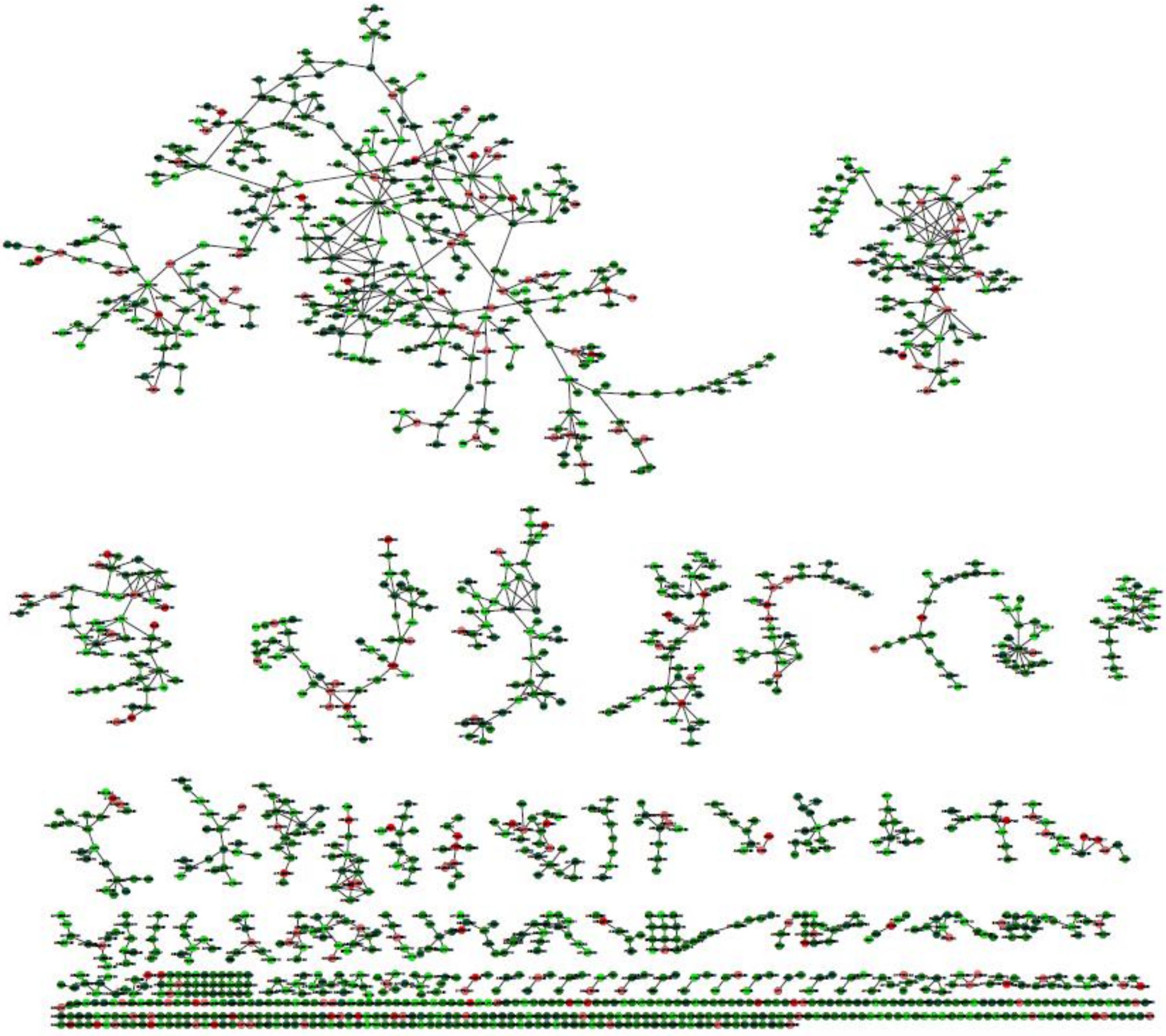

Camalexin Networks at a 0.99 threshold

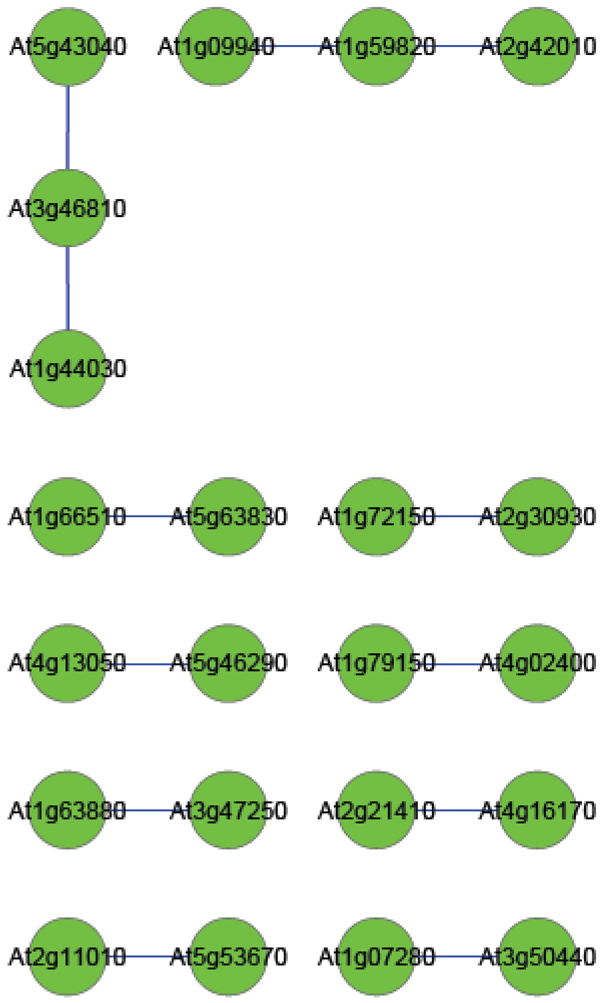

Camalexin Networks at a 0.999 threshold

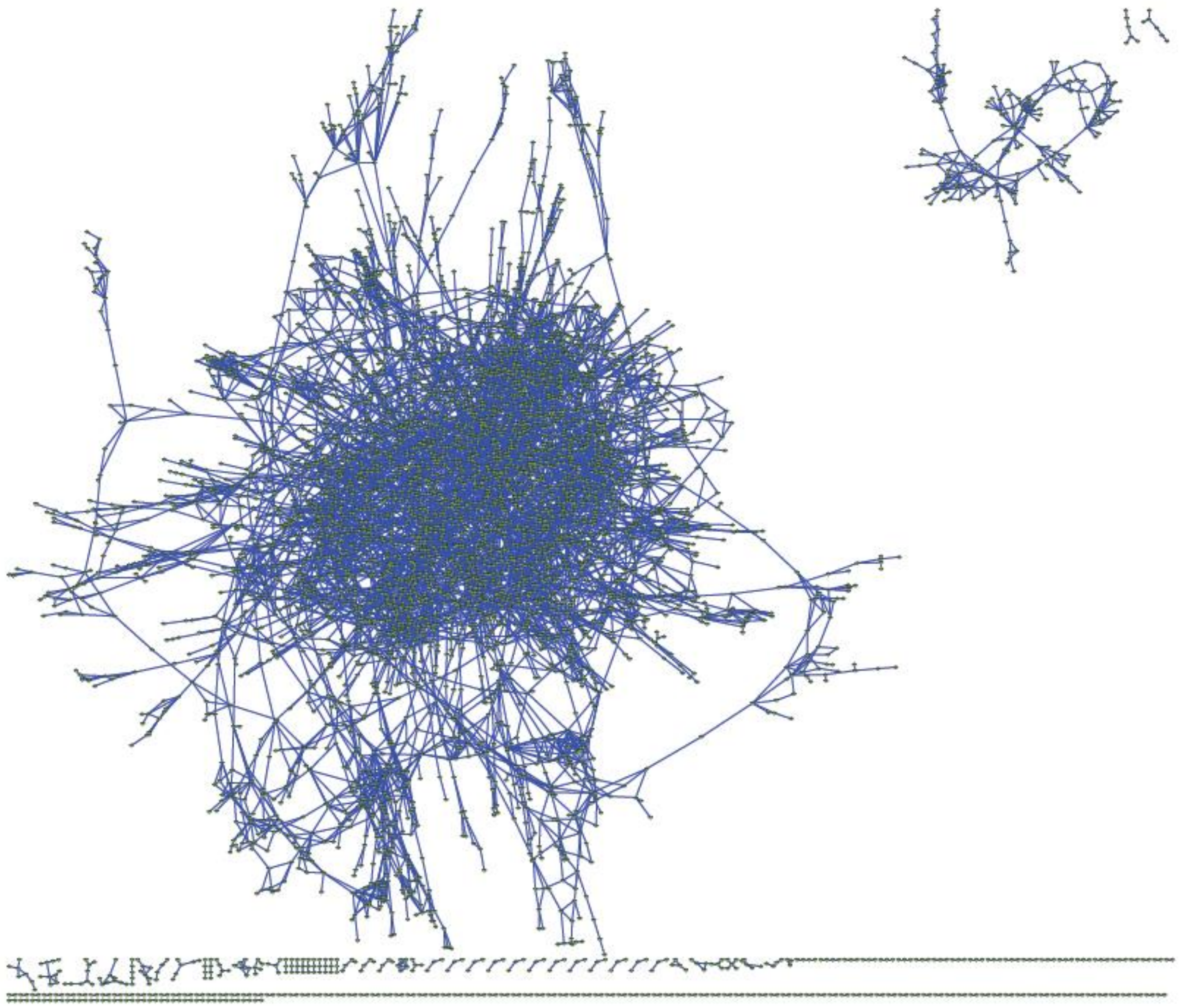

Lesion Area Networks at a 0.95 threshold

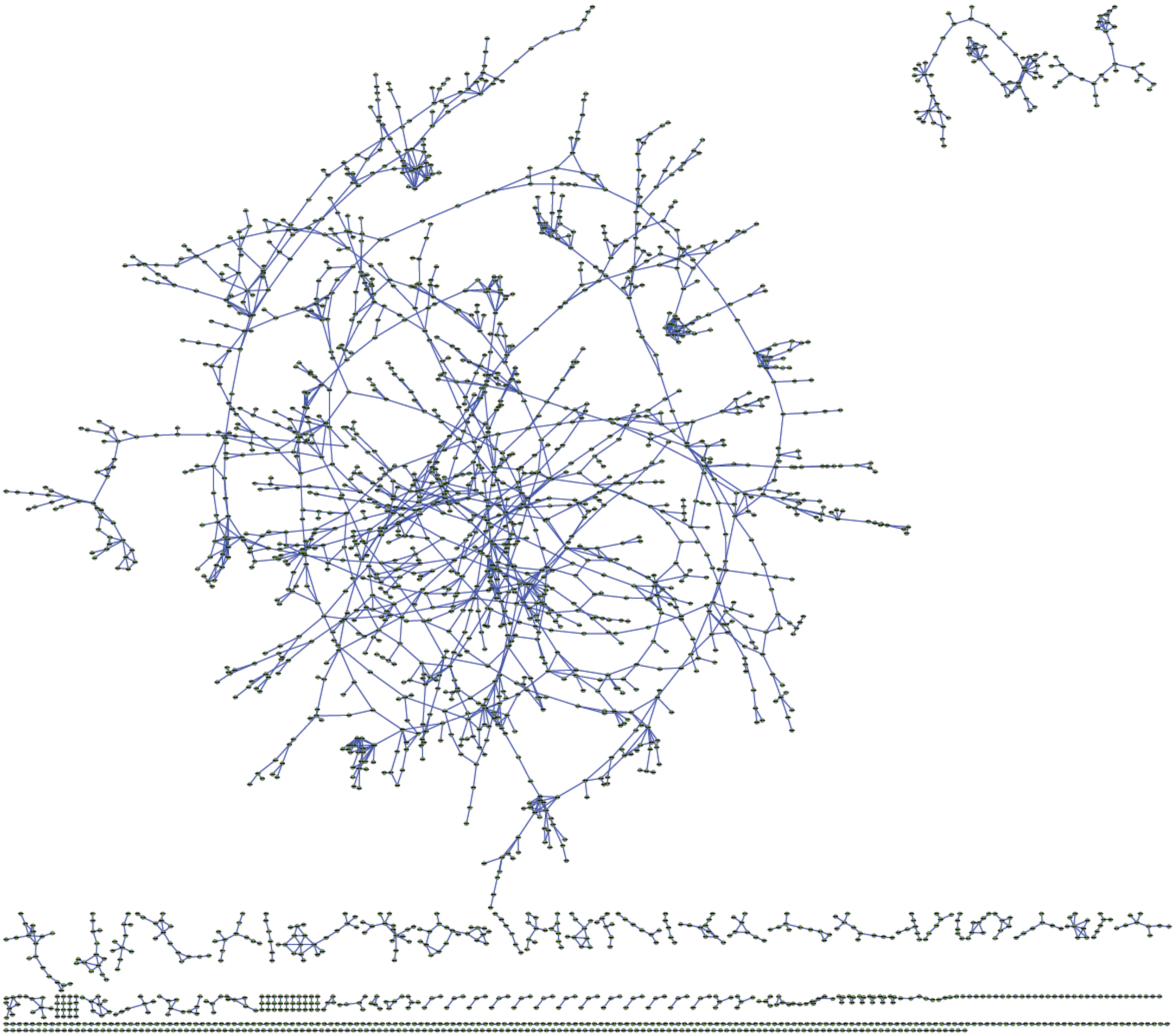

Lesion Area Networks at a 0.975 threshold

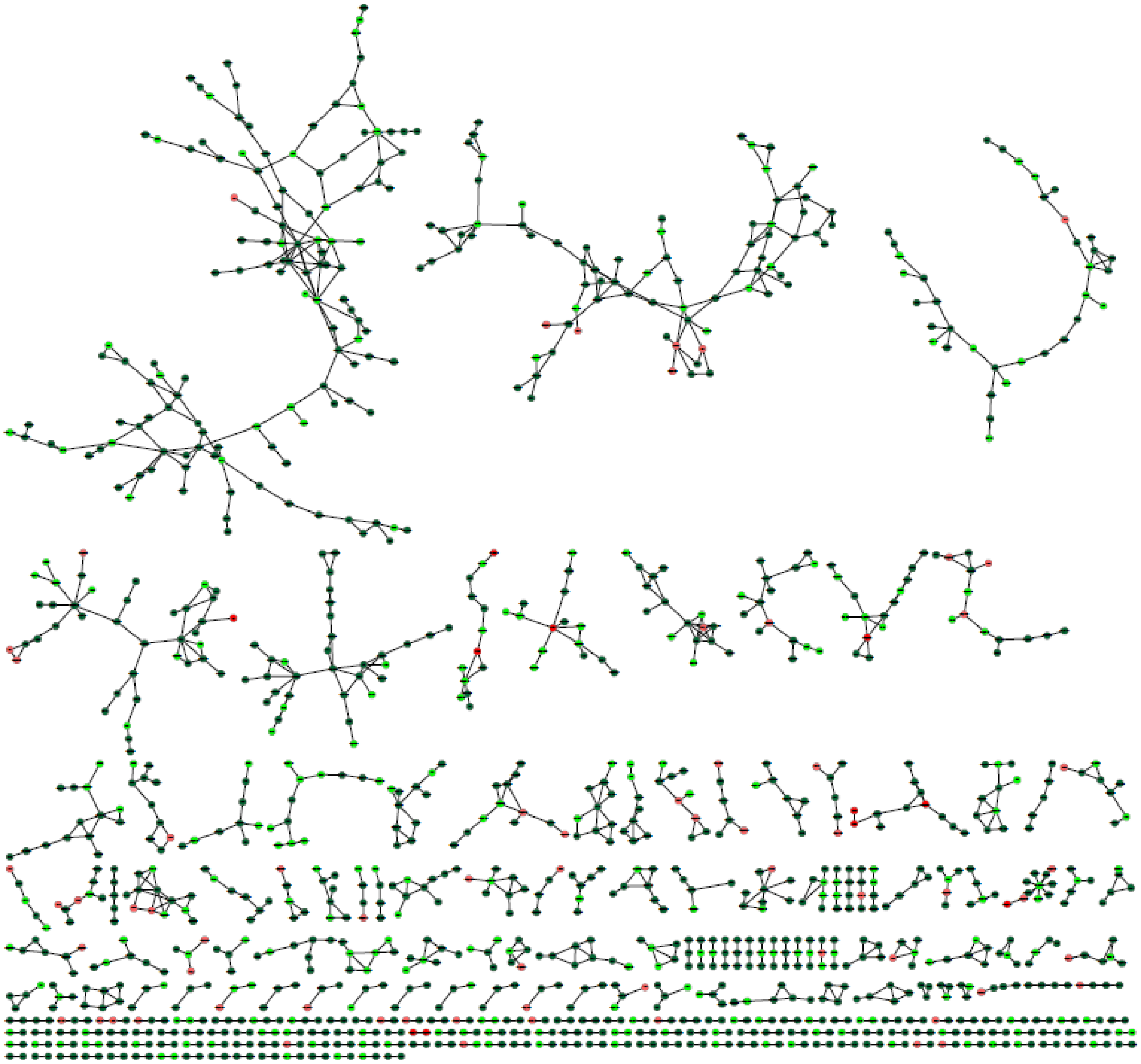

Lesion Area Networks at a 0.99 threshold

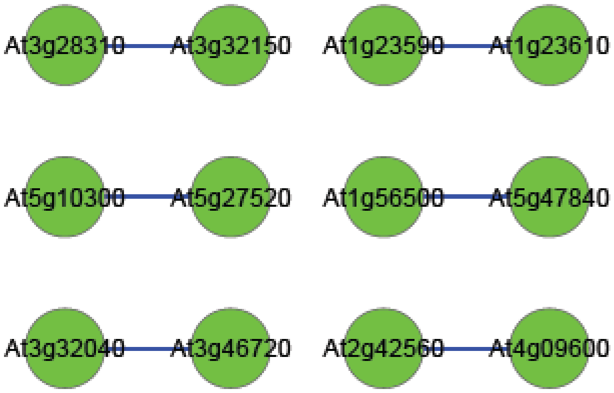

Lesion Area Networks at a 0.999 threshold
